# circCsnk1g3- and circAnkib1-regulated interferon responses in sarcoma promote tumorigenesis by shaping the immune microenvironment

**DOI:** 10.1038/s41467-022-34872-8

**Published:** 2022-11-25

**Authors:** Roberta Piras, Emily Y. Ko, Connor Barrett, Marco De Simone, Xianzhi Lin, Marina T. Broz, Fernando H. G. Tessaro, Mireia Castillo-Martin, Carlos Cordon-Cardo, Helen S. Goodridge, Dolores Di Vizio, Mona Batish, Kate Lawrenson, Y. Grace Chen, Keith Syson Chan, Jlenia Guarnerio

**Affiliations:** 1 Department of Radiation Oncology, Samuel Oschin Comprehensive Cancer Institute, Cedars-Sinai Medical Center, Los Angeles, CA USA; 2 Department of Medical and Molecular Sciences, University of Delaware, Newark, DE USA; 3 Women’s Cancer Research Program at Samuel Oschin Comprehensive Cancer Institute, Cedars-Sinai Medical Center, Los Angeles, CA USA; 4 Division of Gynecologic Oncology, Department of Obstetrics and Gynecology, Cedars-Sinai Medical Center, Los Angeles, CA USA; 5 Department of Pathology, Mount Sinai School of Medicine, The Mount Sinai Medical Center, New York, NY 10029 USA; 6 Board of Governors Regenerative Medicine Institute, Cedars-Sinai Medical Center, Los Angeles, CA USA; 7 Research Division of Immunology, Department of Biomedical Sciences, Cedars-Sinai Medical Center, Los Angeles, CA USA; 8 Department of Surgery and Department of Pathology, Samuel Oschin Comprehensive Cancer Institute, Cedars-Sinai Medical Center, Los Angeles, CA USA; 9 Center for Bioinformatics and Functional Genomics, Samuel Oschin Comprehensive Cancer Institute, Cedars-Sinai Medical Center, Los Angeles, CA USA; 10 Department of Immunobiology, Yale University School of Medicine, New Haven, CT USA; 11 Department of Pathology, Samuel Oschin Comprehensive Cancer Institute, Cedars-Sinai Medical Center, Los Angeles, CA USA; 12 David Geffen Medical School, Department of Hematology Oncology, UCLA, Los Angeles, CA USA

**Keywords:** Cancer microenvironment, Sarcoma, Non-coding RNAs

## Abstract

Exonic circular RNAs (circRNAs) produce predominantly non-coding RNA species that have been recently profiled in many tumors. However, their functional contribution to cancer progression is still poorly understood. Here, we identify the circRNAs expressed in soft tissue sarcoma cells and explore how the circRNAs regulate sarcoma growth in vivo. We show that circCsnk1g3 and circAnkib1 promote tumor growth by shaping a pro-tumorigenic microenvironment, possibly due to their capabilities to regulate tumor-promoting elements extrinsic to the tumor cells. Accordingly, circCsnk1g3 and circAnkib1 can control the expression of interferon-related genes and pro-inflammatory factors in the sarcoma cells, thus directing immune cell recruitment into the tumor mass, and hence their activation. Mechanistically, circRNAs may repress pro-inflammatory elements by buffering activation of the pathways mediated by RIG-I, the cytosolic viral RNA sensor. The current findings suggest that the targeting of specific circRNAs could augment the efficacy of tumor and immune response to mainstay therapies.

## Abstract (web summary)

Circular RNAs can contribute to tumour progression. Here the authors show that two circular RNAs, circCsnk1g3 and circAnkib1 promote sarcoma growth by controlling the expression of interferon related genes and pro-inflammatory factors in sarcoma cells

## Introduction

Circular RNAs (circRNAs) are a new class of predominantly non-coding RNAs that have been recently profiled in many tumors^1–4^. The majority of the identified circRNAs result from alternative splicing events in which the 3’-tail of an exon backsplices and joins with the 5’-head of an exon localized up-stream^5^. Although some transcripts exist exclusively in circular form^6,7^, most circRNAs share a pre-mRNA precursor with their linear transcript counterpart (linRNA)^5,8^. Therefore, the same genetic unit can produce distinct linear and circular RNA isoforms, which have frequently been shown to exhibit independent functions^9,10^. Mounting evidence suggests that circRNAs could contribute to normal and pathological cellular activities; some publications showed for instance that circRNAs regulate the expression of target genes post-transcriptionally by functioning as miRNA decoys^11^. More recently, newer investigations showed the involvement of circRNAs in controlling signaling pathways that ultimately lead to immune activation^10,12^. Accordingly, some circRNAs have been reported to manipulate the immune machinery, possibly enabled by their structural similarities with viral RNAs^10^. This functional mode of circRNAs has been profiled in antiviral immunity and autoimmune diseases. However, whether circRNAs can regulate antitumor immune responses is still poorly understood.

Soft-tissue sarcomas (STS) are tumors of heterogeneous histology that originate from cells belonging to the mesenchymal lineages. Multiple subtypes of STS exist, based on the genetics and differentiation status of the tumor cells^13,14^. For instance, those characterized by numerous genetic aberrations are defined as complex-karyotype sarcomas^13,15^. Among them, Undifferentiated Pleiomorphic Sarcoma (UPS) is one of the most recurrent and aggressive sarcoma subtypes in adults^16,17^. The overall survival of UPS patients has shown only slight improvement in recent decades. Surgery, radiotherapy, and chemotherapy are still the mainstay treatments; however, tumors frequently relapse, showing recurrence at the primary site or in distant organs such as lung. Novel therapeutic interventions, including immunotherapy (e.g., immune-checkpoint inhibitors targeting PD1/PDL1^18^), which potentiates antitumor immune responses, have only provided marginal benefits for these patients so far^19,20^. In this respect, STS are especially immune-excluded tumors^21^; therefore, therapeutic interventions aimed at recruiting antitumor immune cells to the sarcoma mass may be essential to eradicate the disease. Thus, sarcomas represent a tumor type for which it is essential to develop novel therapies that can elicit clinically meaningful and sustained antitumor responses.

Here, we investigated the involvement of circRNAs in STS progression, with a specific focus on whether circRNAs expressed within sarcoma cells can extrinsically influence the composition of the immune tumor microenvironment and trigger anti-sarcoma immune responses. Interestingly, we observe that circRNAs promote sarcoma growth by limiting the expression of interferons and pro-inflammatory cytokines in tumor cells. This action impedes the recruitment and activation of immune cells within the tumor mass, and consequently facilitates the formation of a tumor-promoting microenvironment. Mechanistically, these functions may partially depend on circRNAs’ capability to modulate the activity of the cytosolic RNA sensor RIG-I in the tumor cells. The current findings show a mechanistic aspect of the role of circRNAs in promoting tumor growth, and at the same time provide critical insights on the immune composition of the sarcoma microenvironment, which has not yet been fully characterized.

## Results

### CircAnkib1 and circCsnk1g3 promote sarcoma growth in vivo

The majority of circRNAs exhibit tissue-specific patterns of expression^22^, and are frequently expressed at lower levels compared to their linear counterparts, although the circularized structures prolong their half-life compared to messenger RNAs^23^. We first aimed to identify the most abundantly expressed circRNAs in human and mouse sarcoma cells. Accordingly, we depleted ribosomal RNA from total RNA and performed sequencing to profile the circRNAs expressed in human Undifferentiated Pleiomorphic Sarcoma (UPS) samples (*n* = 8) as well as our recently reported and characterized UPS mouse model^24^ (Supplementary Fig. 1A). By identifying reads that spanned exon boundaries in a non-linear order, we detected thousands of putative sites for circRNA-generating backsplices (Supplementary Data 1, 2). Reasoning that a conservative filtering of these presumed circRNA-generating genes would yield bonafide circRNAs that are best poised to exhibit functional capacity, we narrowed down the number of circRNA-generating loci for further functional characterization. Accordingly, circRNAs were filtered based on high average expression levels (read counts of the backsplice junction and RTqPCR validation), prevalence across human patients, conservation across species, and cross-identification in previous reports or databases^3,25^, identifying 12 circRNA-generating loci meeting these stringent selection criteria (see Methods).

To confirm expression and characterize the functional role of the circRNAs in sarcoma, we employed a syngeneic sarcoma mouse model that would simultaneously (i) recapitulate the genetic aberrations found most frequently in patients, and (ii) provide an immune-competent setting appropriate for studying in vivo the immune microenvironment. By genetic manipulation of the *Trp53* and *Ccne1* genes in mesenchymal stem cells, we generated p53^KO^Ccne1^+^ cells, which were also labeled with a fluorescent protein for tracing (dsRED). We previously reported and deployed this modeling platform, generating tumors that displayed histological features, such as numerous mitotic figures and fibroblastic and pleiomorphic tumor cells, typical of human UPS^24^. Unlike most extant mouse models of STS, this model further recapitulates the human disease in that its genetics reflect the functional loss of *TP53* and gain of *CCNE1*, which were identified among the most frequently occurring genetic and transcriptional defects in the complex-karyotype soft-tissue sarcomas (*The TCGA Research Network, Cell 2017*).

**Fig. 1:**
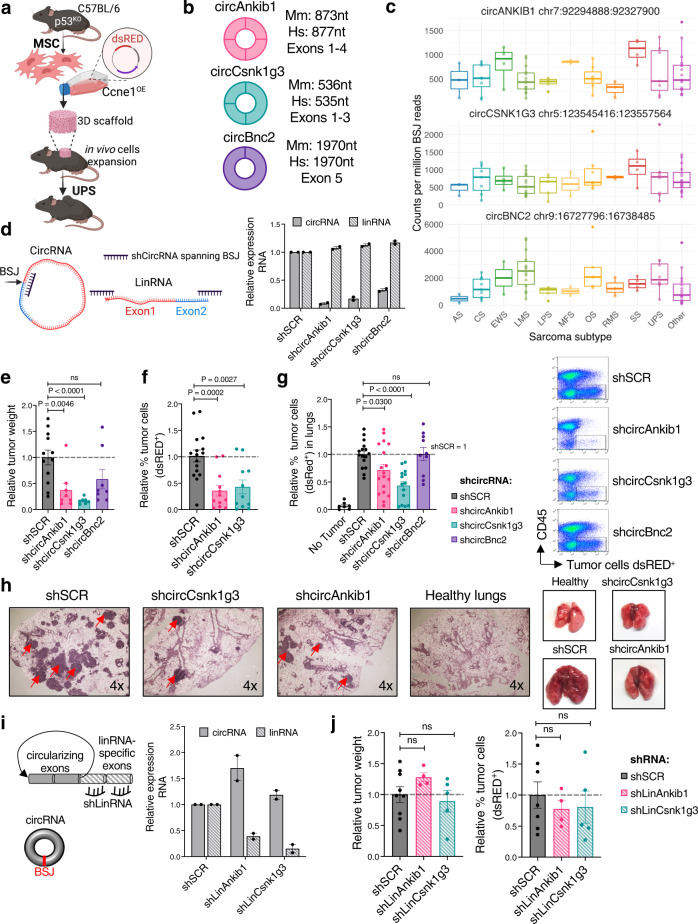
circCsnk1g3 and circAnkib1 promote tumor growth in vivo. **a** Schematic representation of the methodology used to generate dsRED^+^ p53^KO^Ccne1^+^ sarcoma cells from mouse mesenchymal stem cells, and seeding of subcutaneous tumors. **b** Schematic representation of the 3 abundantly expressed, conserved circRNAs. Exons included in the circular transcript and length of the transcript are indicated. **c** Expression of circANKIB1, circCSNK1G3, and circBNC2 in clinical samples of sarcoma subtypes, expressed as counts per million backsplice reads. Subtypes are AS, angiosarcoma (*n* = 3); CS, chondrosarcoma (*n* = 7); EWS, Ewing sarcoma (*n* = 4); LMS, leiomyosarcoma (*n* = 12); LPS, liposarcoma (*n* = 5); MFS, myxofibrosarcoma (*n* = 2); OS, osteosarcoma (*n* = 6); RMS, rhabdomyosarcoma (*n* = 3); SS, synovial sarcoma (*n* = 3); UPS, undifferentiated pleiomorphic sarcoma (*n* = 7); and Other (*n* = 15). Data are presented as median, 25th and 75th percentiles (box), ±1.5 × IQR (whiskers); dots represent individual patient samples. **d** Expression measured by RTqPCR of circular and linear transcripts after targeting of the circular isoforms by using shCircRNAs against the backsplice junction (BSJ). Expression levels are relative to the shSCR condition. Technical replicates are shown from one representative experiment of *n* = 2. **e** Weight of subcutaneous tumors generated by control sarcoma cells (shSCR, *n* = 12 tumors) or from sarcoma cells in which a circRNA was silenced (shcircAnkib1, *n* = 8; shcircCsnk1g3, *n* = 7; or shcircBnc2, *n* = 8). The weight of each tumor is normalized by the average of the control group, over *n* = 2 independent experiments. **f** Relative percentage of tumor cells (dsRED^+^ cells) in the subcutaneous tumors expressing shSCR (*n* = 16) or in which the candidate circRNAs were silenced (shcircAnkib1, *n* = 12; shcircCsnk1g3, *n* = 11). Data are normalized on the average of the control group (shSCR = 1, dotted line, over three independent experiments). **g** Left: relative percentage of tumor cells (dsRED^+^ cells) in the lung, for tumors expressing shSCR (*n* = 16) or tumors in which candidate circRNAs were silenced (shcircAnkib1, *n* = 18; shcircCsnk1g3, *n* = 14; shcircBnc2, *n* = 10). The data are normalized on the average of the control group (shSCR = 1, dotted line), over *n* = 3 independent experiments for shcircCsnk1g3, *n* = 4 independent experiments for shcircAnkib1 and *n* = 2 independent experiments for shcircBnc2. Right: representative flow cytometry gating of the tumor cells (dsRED^+^) and immune cells (CD45^+^) in the tumor mass. **h** Representative H&E staining of one mouse lung section for each experimental group containing tumor foci, indicated by arrows. **i** Schematic representation of the shRNAs used to silence the linear transcripts but not the circRNAs (shLinRNAs, left) and measurement of linear and circular transcripts in qPCR by using convergent and divergent primers (right). *n* = 2 technical replicates are shown from one representative experiment of 2. **j** Weights (left) and percentage of dsRed^+^ tumor cells (right) from subcutaneous tumors generated by control sarcoma cells (shSCR, *n* = 9) or from sarcoma cells in which the expression of linear *Ankib1* or *Csnk1g3* was silenced (*n* = 4 and *n* = 5, respectively). Both weights and percentages are normalized by the mean of the control group (shSCR = 1, dotted line). For all figures, data are reported as mean ± s.e.m. and dots represent independent mice. *P* values: two-tailed unpaired Student’s *t* test. Source data are provided as Source Data File.

After generating transformed p53^KO^Ccne1^+^ cells, these cells were seeded on a biologically inert 3D scaffold and transplanted subcutaneously in syngeneic recipient mice (Fig. 1a). To ensure that the circRNAs identified from RNA-seq were expressed in the sarcoma cells, as opposed to the cells of the tumor microenvironment, we isolated the dsRED-labeled sarcoma cells from growing tumors and assessed the circRNAs’ expression by reverse transcription (RTqPCR) of the RNA ex vivo (Supplementary Fig. 1B). After confirming the expression of 12 candidate circRNAs specifically in sarcoma cells, we assessed whether any of these candidates were higher expressed in malignant cells (p53^KO^Ccne1^+^) compared to analogous, non-tumorigenic cells (p53^KO^ mesenchymal cells)^24^. While all 12 circRNAs trended toward higher average expression in sarcoma cells compared to non-malignant mesenchymal cells, 6 of these—circCamsap1, circRad23b, circBnc2, circRere, circAnkib1, and circCsnk1g3—were significantly upregulated in sarcoma (Supplementary Fig. 1C). Importantly, these 6 circRNAs demonstrated features of bonafide circRNAs: (i) by resisting degradation by RNase R, a 3’−5’ endonuclease able to degrade linear but not circular transcripts (Supplementary Fig. 1D, E)^11^, and (ii) by showing longer half-life than the linear transcripts after Actinomycin D-induced transcription blockade (Supplementary Fig. 1F). Of these, we selected the three top circRNAs whose human analogs were most abundantly expressed in primary patient samples, as assayed by RTqPCR (Supplementary Fig. 1G), for functional studies in the murine model: circAnkib1, circCsnk1g3 and circBnc2 (Fig. 1b). Importantly, the human analogs of these three circRNAs have been detected not only in UPS but in other subtypes of soft-tissue sarcoma (Fig. 1c) and multiple epithelial cancers (Supplementary Fig. 1H) surveyed in the MiOncoCirc database for human primary tumors^3^. In addition, they were abundantly expressed in multiple human sarcoma and carcinoma cell lines (Supplementary Fig. 1I).

For each of these circRNAs, we designed lentiviral vectors expressing shRNAs that target the backsplice junction of the circRNAs to deplete them. Importantly, these shRNAs (indicated as shCircRNAs henceforth) were specifically designed to hybridize only to the sequence of the backsplice junction, while leaving the linear transcripts unaffected (Fig. 1d, left panel). The specific and efficient knockdown of the circRNAs was indeed confirmed by qPCR of shCircRNA vs shSCR sarcoma cells (Fig. 1d, right panel), indicating a decrement in circular but not linear RNA expression in the shCircRNA cells. As these circRNAs exhibited significant upregulation in malignant cells, we hypothesized that they might impinge on the tumorigenic process. To assess this in vivo, the p53^KO^Ccne1^+^ sarcoma cells, in which the expression of circAnkib1, circCsnk1g3, or circBnc2 was silenced, were transplanted in syngeneic immune-competent mice to generate sarcomas either subcutaneously or in the lung (Supplementary Fig. 1J). The subcutaneous tumors were harvested when the control group reached ~1 cm^3^ in size, and the lung tumors after 20 days. Remarkably, when circCsnk1g3 and circAnkib1 were silenced within sarcoma cells, the subcutaneous tumors showed significantly reduced size (Fig. 1e), as well as a lower percentage of dsRED+ tumor cells within the tumor mass, compared to the controls (Fig. 1F). Moreover, when the same circCsnk1g3- and circAnkib1-silenced sarcoma cells were intravenously injected into the tail vein, they exhibited significantly reduced tumor cells and tumor foci number in the lung (Fig. 1g, h). In contrast, the silencing of circBnc2 only marginally affected the growth of the sarcoma cells subcutaneously (Fig. 1e), and it did not affect the tumor burden in the lung (Fig. 1g). While the silencing of circAnkib1 and circCsnk1g3 showed a profound effect on tumor growth in vivo, the silencing of linear *Ankib1* and *Csnk1g3* transcripts with shRNAs specific to the linear isoforms (Fig. 1i) did not impact tumor growth (Fig. 1j). These results reinforce the findings that linear and circular transcripts from the same gene can play independent roles in cancer despite partially shared nucleotide sequence. Moreover, these data highlighted the tumor-promoting roles of circCsnk1g3 and circAnkib1 in the context of sarcoma, warranting further mechanistic investigations.

### circAnkib1 and circCsnk1g3 regulate the expression of interferons and pro-inflammatory signals in the sarcoma cells through RIG-I-mediated pathways

**Fig. 2:**
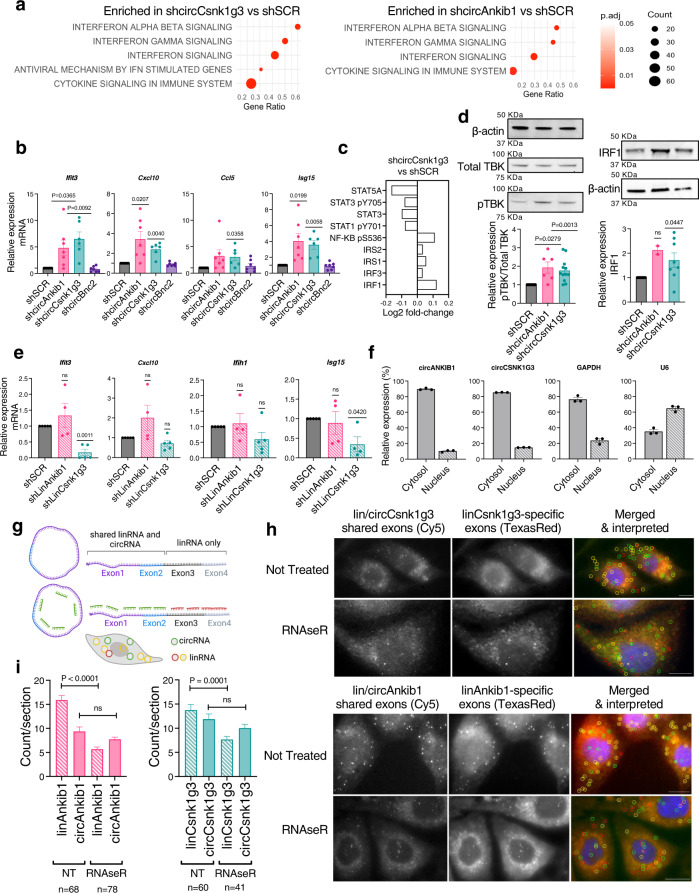
circRNAs regulate the expression of interferon signals and pro-inflammatory cytokines in tumor cells. **a** Pathway analysis showing top Reactome terms enriched upon knockdown of circCsnk1g3 (left) or circAnkib1 (right). Pathways are colored by Benjamini-Hochberg adjusted *P* value. **b** Validation by RTqPCR of the differential expression of interferon-related genes after silencing of circCsnk1g3, circAnkib1, and circBnc2. Dots represent *n* = 6 independent biological replicates (minimum) and *P* values are derived from two-tailed one sample *t* test. **c** RPPA analysis of specific regulators of interferon signaling. **d** Validation and quantification of changes in pTBK (left) and IRF1 (right) expression in sarcoma cells after silencing of circCsnk1g3 (*n* = 12 pTBK and *n* = 8 IRF1) and circAnkib1 (*n* = 6 pTBK and *n* = 2 IRF1). Dots represent independent biological replicates, and one representative western blot is shown for each protein. *P* values derived from two-tailed one sample *t* test. **e** RTqPCR analysis of the differential expression of interferon-related genes after the silencing of the linear (mRNA) isoforms of *Csnk1g3* and *Ankib1*. Dots represent *n* = 4 independent biological replicates (minimum) and *P* values are derived from two-tailed one sample *t* test. **f** Fractionation of nucleus/cytosol and quantification of circCsnk1g3 in the two distinct fractions. *Gapdh* (cytosolic) and *U6* (nuclear) were used to validate the fractionation. Dots represent technical replicates of one experiment. **g** Schematic representation of the circFISH assay used to distinguish linear and circular isoforms and to map their sub-cellular localization. Using fluorescent probesets targeted to specific exons, the shared exons (linRNA and circRNA) were labeled with Cy5 (green) and the linRNA-only exons were labeled with Texas Red, such that linear transcripts appear as red or yellow signal, and circRNAs as green. **h** Representative pictures of the circFISH assay to detect circular and linear isoforms of *Csnk1g3* and *Ankib1*, with overlaid circles in rightmost panels denoting detection of a linRNA (yellow or red circle) or circRNA (green circle). Top panels: untreated sarcoma cells. Lower panels: sarcoma cells treated with RNAseR. Representative pictures are shown from *n* ≥ 2 independent experiments. **i** Quantification of the circFISH assay for circCsnk1g3, linCsnk1g3 and circAnkib1, linAnkib1 in the RNaseR and untreated (NT) conditions. *P* values are derived from unpaired Student’s *t* test. For all bar plots, data are reported as mean fold-change ± s.e.m. unless otherwise noted. Source data are provided as Source Data File.

How do circCsnk1g3 and circAnkib1 promote sarcoma growth? To answer this question, we investigated the tumor-related pathways regulated by these circRNAs in the sarcoma cells. Accordingly, we silenced the expression of circAnkib1 and circCsnk1g3 in the tumor cells using shCircRNAs (as in Fig. 1d), and then analyzed the cells’ transcriptomic profiles by RNA sequencing. Gene Set Enrichment Analysis demonstrated upregulation of signaling pathways related to both the production of and response to type-I and type-II interferons, following knockdown of both circAnkib1 and circCsnk1g3 (Fig. 2a). Consistently, we found increased expression of interferon-related genes (e.g., *Ifit3*, *Isg15*, *Ifih1*, *Oasl2*, and *Irf7*) and pro-inflammatory cytokines (e.g., *Cxcl10*, *Cxcl9*, *Ccl3*, and *Ccl5*) in the tumor cells upon circAnkib1 and circCsnk1g3 silencing; these results were validated by RTqPCR for selected genes (Fig. 2b). We tested an additional 2 independent shRNA designs against each circRNA, and observed the same upregulation in interferon-response and cytokine-encoding genes (Supplementary Fig. 2A, B). To test whether these observations could be corroborated at the protein level, we employed Reverse Phase Protein Arrays (RPPA) on the circCsnk1g3-silenced cells, followed by western blot validations. In the sarcoma cells silenced for the expression of circCsnk1g3, compared to the controls, the RPPA showed upregulation of NFκB, interferon regulatory factors (IRFs), and the insulin receptor substrates (IRSs), which are engaged by interferon signaling^26^ (Fig. 2c). Interestingly, despite expressing these inflammatory elements, circCsnk1g3-silenced cells expressed less STAT1 than control. STAT1 is known to function as a mediator of autocrine and paracrine interferon signaling, which downstream impinges on the NFκB and IRF pathways. A relative lack of STAT1 expression, therefore, suggests that the activation of inflammatory machinery in circCsnk1g3-silenced cells may originate from intracellular signals; intriguingly, pathway analysis on these data demonstrated upregulation of intracellular viral RNA sensing machinery (Fig. 2a). Meanwhile, control cells expressed more of both STAT3 and STAT5, where the former is a principal mediator of IL-10 and IL-6 induced signaling^27^, and the latter has been associated with suppression of antitumor immunity^28^ and reduction of tumor response to stimulation by IFNα^29^. Additionally, western blot analysis demonstrated that the expression of IRF1 and pTBK, both critical signaling elements for transcription of interferons and inflammatory cytokines, was enhanced in the circCsnk1g3- and circAnkib1-silenced tumor cells, in line with the RNA-level data (Fig. 2d). Importantly, the increased expression of pro-inflammatory pathways were uniquely triggered by circRNA silencing, while the knockdown of the linear transcripts did not show such an effect or even trended in the opposite direction (Fig. 2e and Supplementary Fig. 2C).

Next, we aimed to investigate the molecular mechanisms underlying the capacity of these circRNAs to regulate interferons and pro-inflammatory signals in sarcoma tumor cells. CircRNAs have been reported to activate or suppress elements of innate immunity^10,12,30^. This potential functional mode of circRNAs has been poorly described in tumors, however. Because circAnkib1 and circCsnk1g3 both exhibited anti-inflammatory capacity, we hypothesized that circAnkib1 and circCsnk1g3 could interact with double-stranded RNA (dsRNA)-binding proteins that function as pattern-recognition receptors and/or mediators of antiviral immunity, such as PKR, MDA5, and RIG-I. As these viral RNA-sensing mechanisms are predominantly cytosolic, we first employed nucleus/cytosol fractionation, which indeed ascertained that circAnkib1 and circCsnk1g3 also localized preferentially in the cytosol (Fig. 2f). Cytosolic localization was confirmed by RNA-targeted fluorescence in situ hybridization, using a specially adapted technique to distinguish between circular and linear RNA isoforms (circFISH^31^, Fig. 2g, h). Linear RNA degradation by RNase-R treatment was used to verify the circular-vs-linear specificity of the circFISH signals (Fig. 2i).

Next, we sought to detect interactions between circAnkib1 and circCsnk1g3 to viral RNA sensing proteins. Recent work suggested that protein kinase R (PKR), a nucleic acid receptor that induces a signal transduction cascade ultimately leading to interferon production, can bind endogenous cellular circRNAs in addition to viral RNAs^10^. In the innate-immunity setting, endogenous circRNAs were degraded in the presence of viral RNAs, freeing PKR to trigger interferon expression and immune-activating signals^10^. Thus, we first hypothesized that circCsnk1g3 and circAnkib1 could function as negative regulators of PKR in the tumor cells, and thereby block the activation of interferon signals. We accordingly generated CRISPR/Cas9 knockout (KO) sarcoma cells for PKR (Supplementary Fig. 3A) and measured the capability of the circRNAs to trigger inflammatory signals in these cells, compared to in PKR-WT (control) cells. In PKR-WT cells, silencing of circCsnk1g3 and circAnkib1 were both capable of triggering the expression of inflammatory elements as expected (Supplementary Fig. 3B). However, this capability was not significantly decreased in the PKR-KO setting (Supplementary Fig. 3B), suggesting that, in the tumor context, the circRNAs do not necessarily depend on PKR to mediate their pro-inflammatory effect.

**Fig. 3:**
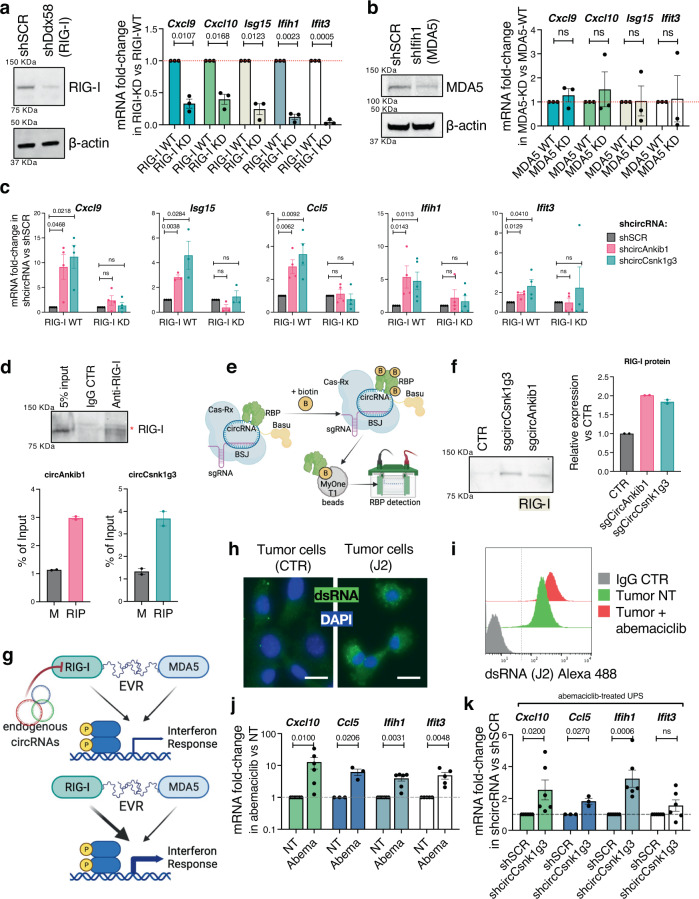
circRNAs regulate the expression of interferon genes partially through RIG-I. **a** Left: validation of stable RIG-I knockdown by shDdx58 in UPS cells. Right: RTqPCR quantification of interferon-related genes, in control cells (RIG-I WT, *n* = 3) or in RIG-I-silenced cells (RIG-I KD, *n* = 3). Dots represent biological replicates. **b** Left: validation of stable MDA5 knockdown by shIfih1 in UPS cells. Right: RTqPCR quantification of interferon-related genes, in the control cells (MDA5 WT, *n* = 3) or in MDA5-silenced cells (MDA5 KD, *n* = 3). Dots represent biological replicates. **c** RTqPCR quantification of shcircRNA-mediated changes in interferon-related gene expression, in RIG-I WT or RIG-I KD sarcoma cells. Dots represent biological replicates. **d** Top: validation of the RIG-I pulldown efficiency for RNA immunoprecipitation. 5% of total material is probed in Input, the remaining 95% was split evenly for the IgG and RIG-I pulldowns. One representative blot of 2 is shown. Bottom: detection of circRNAs in Mock (M) or RIG-I (RIP) immunoprecipitation, as percent of input. Dots represent technical replicates of one representative experiment out of 3 with similar trend. **e** Schematic of the BASU-dCasRx system for detecting interaction between circRNA and protein. **f** Left: western blot of RIG-I protein detected upon circRNA-guided biotin pulldown. One representative blot of 2 is shown. Right: Quantification of same. Dots represent technical replicates from one representative experiment of two with similar trend. **g** Schematic representation of the working hypothesis for circRNA impingement on activity of viral RNA sensor RIG-I. **h** Sections of mouse sarcomas with immunofluorescence staining of endogenous dsRNAs by J2 antibody. Two representative sections of one experiment are shown. **i** Flow cytometry quantification of dsRNA content in sarcoma cells, measured by intracellular staining with J2 antibody. One representative histogram from *n* = 3 independent samples is shown. **j** RTqPCR quantification of abemaciclib-mediated changes in interferon-related gene expression in UPS cells. Expression fold-changes normalized to no-treatment condition (NT, dotted line). Dots represent biological replicates from *n* ≥ 3 independent experiments. **k** RTqPCR quantification of interferon-related gene expression, in abemaciclib-treated shcircCsnk1g3 sarcoma cells vs abemaciclib-treated shSCR cells. Fold-changes normalized to shSCR condition (dotted line). Dots represent biological replicates from *n* ≥ 3 independent experiments. **a**–**c**, **j**, and **k**, dots represent biological replicates, and *P* values are derived from two-tailed one sample *t* test. For all figures, data are reported as mean ± s.e.m. Source data are provided as Source Data File.

Besides PKR, several other proteins have a well-established ability to bind viral RNA and trigger interferon and inflammatory responses. Among these proteins are MDA5 and RIG-I, both pattern-recognition receptors. Thus, we investigated whether MDA5 and RIG-I could also sense endogenous circRNAs. First, we assessed the activation status of these proteins in the sarcoma cells by measuring interferon signals upon MDA5 and RIG-I silencing. The knockdown of *Ddx58* (encoding RIG-I) significantly reduced the expression of interferon signals and pro-inflammatory cytokines in the tumor cells (Fig. 3a). On the contrary, the silencing of *Ifih1* (encoding MDA5) only minimally changed the expression of these elements (Fig. 3b). Thus, we investigated whether the circRNAs’ capacity to repress inflammatory signals could depend on RIG-I. We generated RIG-I shRNA-silenced (RIG-I KD) sarcoma cells, and we measured the capability of the circRNAs to regulate inflammatory signaling in RIG-I KD cells compared to in RIG-I wild-type (RIG-I WT) cells. Upon silencing of each of circCsnk1g3 or circAnkib1, interferon-related genes and pro-inflammatory elements were increased in the RIG-I WT cells, as expected (Fig. 3c). However, such an increase was significantly abrogated in the RIG-I KD condition (Fig. 3c). For circCsnk1g3, this experiment was repeated again using an alternative method to silence RIG-I. Employing CRISPR/Cas9 knockout for RIG-I, again the circRNA modulation of inflammatory signaling was abrogated in RIG-I KO cells compared to RIG-I WT (Supplementary Fig. 3C, D).

Since RIG-I is known to directly bind viral RNA, we next sought to determine whether circRNAs could have a physical interaction with RIG-I as well, which might underlie their modulation of RIG-I function. RIG-I has been estimated to be expressed at ~1000 copies per cell at the upper end of expression (in innate immune cells such as macrophages^32^) while circCsnk1g3 and circAnkib1 were expressed in the sarcoma cells at ~2200 and 400 copies per cell, respectively (Supplementary Fig. 3E), which would potentially allow for an approximately equimolar interaction. Upon immunoprecipitation of RIG-I, RTqPCR on the pulled-down material demonstrated enrichment of circCsnk1g3 and circAnkib1 (Fig. 3d, Supplementary Fig. 3F), whereas no enrichment was observed for the linear transcripts, nor for circBnc2 (Supplementary Fig. 3F)—this result was consistent with the observation that circBnc2 does not regulate interferon signals in the sarcoma cells (Fig. 2b), and does not promote tumor growth (Fig. 1e, g).

The interaction between RIG-I and the circRNAs was also assessed by pulldown in the reverse direction, employing an adapted form of CRISPR-assisted RNA-protein interaction detection^33^ (CARPID). Briefly, the catalytic dead CasRx protein fused with the biotin ligase BASU (BASU-dCasRx) was expressed in the sarcoma cells—along with sgRNAs targeting the backsplice junctions of the circRNAs—such that the BASU-dCasRx was guided to add biotin residues to proteins in the proximity of circCsnk1g3 and circAnkib1 (Fig. 3e). Upon biotin pulldown, RIG-I could be detected among the biotinylated proteins that interact with circCsnk1g3 and circAnkib1 (Fig. 3f).

These data suggest that RIG-I is a major promoter of interferon signals and expression of inflammatory elements in the sarcoma cells, and that endogenous circRNAs may reduce its activation. However, one question remains open: what stimulates RIG-I activation at steady-state in the tumor cells, when RIG-I is canonically activated by RNAs of viral origin? Interestingly, while tumor cells are not directly infected by viruses, they can still present high levels of dsRNAs of viral origin that derive from the re-expression of endogenous retroviral elements (ERVs) integrated into normal mammalian genomes^34^. While generally silent in normal cells, ERVs can be aberrantly expressed in tumor cells, ultimately activating interferon responses through cytosolic RNA sensors like RIG-I and MDA5^35^, in a phenomenon described as “viral mimicry”. Not only may tumor cells at baseline express the high levels of dsRNA necessary to trigger the viral mimicry response, but this response can be enhanced further upon treatment with DNA-demethylating agents^35^, including the CDK4/6 inhibitor abemaciclib^36^. Following this line, we investigated the possibility that endogenous dsRNAs are present in the p53^KO^Ccne1^+^ sarcoma cells, that they trigger inflammatory activity, and that endogenous circAnkib1 and circCsnk1g3 limit viral mimicry responses (Fig. 3g). Consistent with these hypotheses, dsRNA was detectable (with the J2 antibody) in the sarcoma cells at steady-state (Fig. 3h), and was elevated further upon abemaciclib treatment (Fig. 3i). Furthermore, in addition to elevating dsRNA expression, treatment with abemaciclib increased also the expression of interferon and pro-inflammatory elements in the sarcoma cells (Fig. 3j). Finally, in abemaciclib treated sarcoma cells, silencing of circCsnk1g3 increased expression of these genes even further (Fig. 3k), demonstrating that targeting circRNAs could augment the effect of abemaciclib on the sarcoma cells’ viral mimicry response, and potentially also on antitumor immunity.

### Silencing of circRNAs affects the immune TME landscape

**Fig. 4:**
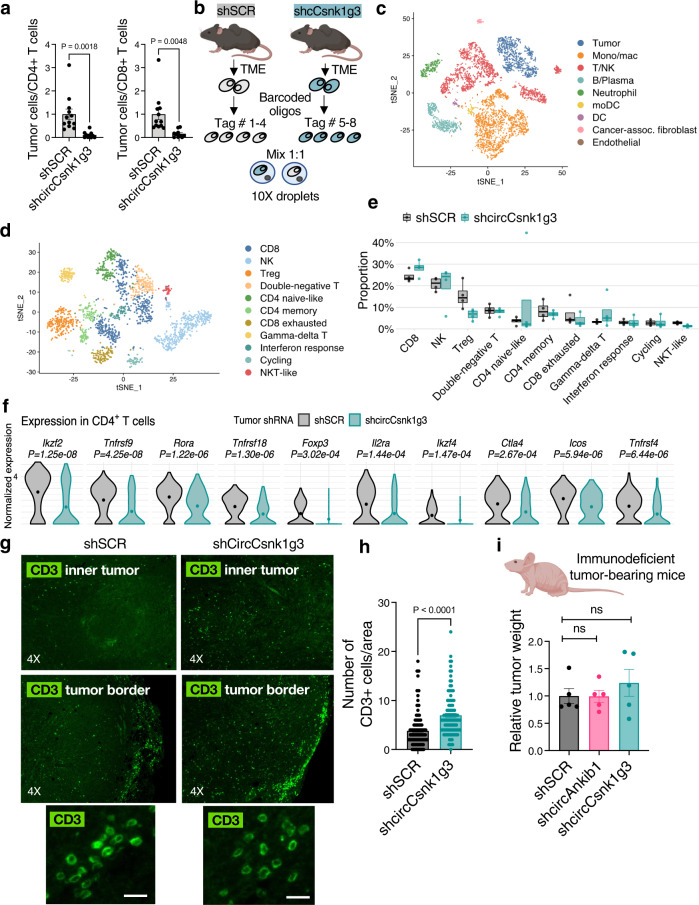
Targeting circRNAs in tumor cells re-shapes the tumor microenvironment. **a** Flow cytometry analysis for ratio between tumor cells and CD4^+^ T cell (left) or CD8^+^ T cells (right) in the subcutaneous sarcoma generated by control sarcoma cells (shSCR, *n* = 12 mice) or cells in which the expression of circCsnk1g3 was silenced (shcircCsnk1g3, *n* = 10 mice). Data are normalized to shSCR condition, over *n* = 3 independent experiments. **b** Schematic representation of the single-cell RNA-sequencing experiment. **c** t-SNE plot of the major cell populations identified. **d** t-SNE plot of lymphocyte subclusters. **e** Comparative proportions of lymphocytic cell identities in shSCR and shcircCsnk1g3 tumors (*n* = 4 mice each group, totaling *n* = 1143 immune cells from shSCR tumors and *n* = 993 immune cells from shcircCsnk1g3 tumors). Data are reported as median, IQR (box), and 1.5 × IQR (whiskers). Dots represent individual mice. **f** Normalized expression of selected genes differentially expressed in CD4^+^ T cells from shSCR and shcircCsnk1g3 tumors, analyzed by single-cell RNA-sequencing. *n* = 273 and *n* = 186 CD4^+^ T cells from *n* = 4 shSCR and *n* = 4 shcircCsnk1g3 tumors, respectively. Mean expression values are plotted as dots and *P* values derived by Wilcoxon rank sum test with Bonferroni correction. **g** Immunofluorescence staining showing the distribution of CD3^+^ T lymphocytes at the border or in the inner tumor parenchyma upon silencing of circCsnk1g3 in the sarcoma cells. Representative pictures of the inner tumor and tumor border areas are shown. **h** Quantification of the total CD3^+^ T cells identified in tumor sections. Dots represent individual regions (*n* = 171 shSCR, *n* = 109 shcircCsnk1g3) acquired from *n* ≥ 2 mice each group. **i** Weight of subcutaneous tumors generated in immunocompromised mice by control sarcoma cells (shSCR, *n* = 5 mice) and by sarcoma cells silenced for expression of circCsnk1g3 (*n* = 5) or circAnkib1 (*n* = 5). Weights normalized to shSCR condition. For all figures, data are reported as mean ± s.e.m., dots represent independent mice, and *P* values determined by unpaired Student’s *t* test, unless otherwise indicated. Source data are provided as Source Data File.

The knockdown of circAnkib1 and circCsnk1g3 resulted in increased interferon and pro-inflammatory signaling in the tumor cells. Tumor derived pro-inflammatory pathways have been widely recognized to play a critical role in tumor development, due to their communication with the immune microenvironment. Therefore, we investigated whether circCsnk1g3 and circAnkib1 may regulate tumor growth by impinging on the overall immune landscape within the tumor microenvironment. To assess this possibility, we performed a comparative analysis of the TME of sarcoma, generated by p53^KO^Ccne1^+^ cells either expressing or silenced for circCsnk1g3. First, by flow cytometry, we surveyed the ratio between tumor and immune cells. The circCsnk1g3-silenced tumors showed a significantly decreased ratio between tumor cells and T cells, both CD4+ and CD8+, compared to the controls (Fig. 4a). Then, to better define the subtypes of immune cells infiltrating these tumors and to characterize their transcriptional changes following from the tumor-cell knockdown of circCsnk1g3, tumors were assayed by droplet-based single-cell RNA-sequencing (scRNA-seq). Subcutaneous sarcomas (shSCR and shCircCsnk1g3, *n* = 4 independently hashtagged mice per group) were dissociated and captured with the Chromium platform (10X Genomics) (Fig. 4b). Following in silico quality control to remove dead cells and doublets, we identified 9 high-level clusters which were detected across conditions, representing tumor cells and the main cell populations of the TME (Fig. 4c, key marker genes shown in Supplementary Fig. 4A and full gene lists in Supplementary Data 4). After annotating the major immune TME subpopulations according to previously published markers^37,38^, we focused on NK and T lymphocytes by separate subclustering (Fig. 4d, Supplementary Data 5). We compared (i) the relative abundance of each cell type in each condition and (ii) changes in the expression profiles of each cell type across conditions. Four populations of CD4+ T cells were distinguished, including regulatory T cells (Treg) expressing *Foxp3, Il2ra, Ikzf2, Tnfrsf4* and *Ctla4*, and CD4+ helper T cells expressing *Ramp3, Rora*, and *Odc1*, genes that have been associated to memory T cells^39^. We identified two clusters of naïve-like CD4+ T cells, expressing *Foxp1* and *Lef1* and either *Ccr7* or *Tcf7*. Cytotoxic CD8+ cells expressing *Gzmk, Cxcr6, Ccl5*, and *Grap2* or the NK receptors *Klra6, Klra7, Klrk1*, and *Klrd1*^40^ were also detected. Additionally, a separate population of CD8+ T cells exhibited exhaustion markers *Lag3* and *Pdcd1*. By comparing the relative abundance of these cell types across conditions, we found that silencing of circCsnk1g3 was associated with a decreasing trend for regulatory T cells (Treg) and exhausted CD8+ T cells; conversely, cytotoxic CD8+ T cells showed the opposite trend (Fig. 4e). Examining differentially expressed genes across conditions, we found that in the tumors silenced for circCsnk1g3 compared to control tumors, CD4+ T cells on average expressed lower levels of Treg-related genes, including *Tnfrsf9, Tnfrsf4, Ikzf2, Il2ra*, and *Ctla4* (Fig. 4f), while CD8+ T cells trended towards lower levels of regulatory/exhaustion genes, including *Pdcd1*, *Ctla4*, *Ikzf2*, and *Tox* (Supplementary Fig. 4b). This was also confirmed with independent cohorts of mice by FACS-sorting CD8+ T cells and RTqPCR analysis of the critical genes (Supplementary Fig. 4C). Importantly, immunofluorescence staining of the sarcoma upon circCsnk1g3 silencing confirmed an increased infiltration of CD3+ T cells in the inner tumor parenchyma of the circRNA-KD tumors, contrasting with the control tumors, in which the T cells mainly accumulated at the tumor borders (Fig. 4g, h). In addition, FoxP3^+^ T regulatory cells were diminished as a fraction of total T cells in the circRNA-KD tumors (Supplementary Fig. 4D, E). These observations reinforce the hypothesis that circRNA silencing could enhance antitumor T-cell responses. Next, to further confirm the involvement of T cells in circRNA-mediated functions, we compared growth of WT and circRNA-silenced sarcoma cells in immunodeficient nude mice that do not bear T lymphocytes. While the silencing of circCsnk1g3 and circAnkib1 reduced tumor growth in immunocompetent mice (as shown in Fig. 1), the silencing of the circRNAs did not affect tumor growth in the immunodeficient mouse strain (Fig. 4i), suggesting that a fully functional immune system, which includes T cells, is necessary for these circRNAs to exert their pro-tumorigenic effect.

Lastly, we characterized the sarcoma-infiltrating myeloid and dendritic cell types (markers in Supplementary Data 6), which included granulocytes (*S100a8*, *Csf3r*), dendritic cells (monocyte-derived *Cd209a*/*Ccr7*, or plasmacytoid *Ccr9*/*Siglech*), and monocytes/macrophages. Non-classical monocytes (*Ace*, *Adgre4*, *Itgax*, *Cx3cr1*) and classical monocytes (*Ccr2*, *Ly6c2*) were present, as were intermediate monocytes expressing lower levels of *Ly6c2* and *Ccr2* along with *Spn* (encoding CD43), but high levels of *Sell* (encoding CD62L). Another myeloid population exhibited an inflammatory intermediate state between monocytes and macrophages including low levels of both *Ly6c2* and *Adgre1*, but high expression of antigen-presentation- and interferon-related genes. Among macrophages, most cells expressed high levels of complement (*C1qa*, *C1qb*, *C1qc*), which in tumor-associated macrophages have been reported to correlate with immune exhaustion and poor survival^41^. The remainder of the macrophages, on the contrary, expressed *Spp1* and *Cd36*, with a distinct subset of these cells also expressing genes related to lipid processing (*Lipa*, *Fabp5*, *Lpl*). Recently, the *1* macrophage phenotype has been reported in human tumors across distinct histology and has been associated with signatures of hypoxia (*Hilpda*, *Hmox1*, *Bnip3*) and lipid metabolism^42^. Tumors silenced for the expression of circCsnk1g3 showed lower infiltration of complement-expressing macrophages but more *Spp1*-expressing macrophages (Supplementary Fig. 4F, G), suggesting that the different inflammatory microenvironments primed by the tumor cells may be responsible for a metabolic switch in the activation of the myeloid cells.

Together these data showed that silencing of circCsnk1g3 in sarcoma cells significantly changed the overall TME immune landscape—in terms of relative numbers of cell populations, their transcriptional activity, and infiltration of T cells beyond the tumor border—to less tumor-permissive conditions that could potentially facilitate the observed reduction in tumor growth.

## Discussion

Previous experimental settings have shown that circRNAs could play pro-tumorigenic roles by regulating mechanisms intrinsic to tumor cells, such as the expression of genes that promote the tumor cell cycle and proliferation. In the context of prostate cancer, circCsnk1g3 has been shown to modulate tumor cell proliferation through the interaction with miR-181b/d and the consequent regulation of cell-cycle related genes (e.g., *CDK1* and *CDC25*)^1^. In addition, circAnkib1 has been reported to be upregulated in osteosarcoma compared to normal and adjacent tissues. In that context, circAnkib1 silencing represses tumor progression by upregulating miR-217 and downregulating *PAX3*^43^. While these and other past publications have reported that circRNAs function as decoys of micro RNAs (miRNAs) in the tumor cells, our data add a different layer of complexity to the mechanisms used by circRNAs to promote tumor growth. Here, we show that circRNAs could repress pathways that drive interferons and pro-inflammatory cytokines in the tumor cells and in this way be ultimately responsible for recruiting antitumor immune activity to the tumor microenvironment. While circRNAs have been reported to modulate interferon signaling in the context of viral infection and autoimmune diseases^10^, similar roles have not been reported in the context of tumor progression.

This study shows that silencing specific circRNAs, including circCsnk1g3 and circAnkib1, removes the circRNA-mediated blockade of interferons and inflammatory cytokines in the tumor cells and thus increases tumor immunogenicity. Such a situation may also have important clinical implications. Tumors poorly infiltrated by immune cells are unlikely to respond to existing immunotherapies based on immune-checkpoint blockade^44^. In this respect, the therapeutic targeting of circRNAs could be explored as adjuvant therapy in combination with other immunotherapeutic approaches—such as anti-PD1/PDL1 immune-checkpoint inhibitors—that are typically precluded in cases of poor preexisting immune infiltration. Furthermore, in this model, T cells infiltrating tumors lacking circCsnk1g3 showed reduced exhaustion markers, an indicator for possible synergy with immune-checkpoint inhibitors, which indeed function to reinvigorate exhausted T cells in the tumor mass^45^. Additionally, targeting of circRNAs could be tested in combination with more conventional cancer treatments, such as radiotherapy or DNA-demethylating agents, to alter the immune composition and function of the TME^46^. Accordingly, our data showed that circRNAs could be critical players in the context of viral mimicry-like responses induced by abemaciclib^36^. In this context, circRNAs could potentially be restricting the drug’s efficacy by limiting the full activation of interferon and inflammatory responses. Thus, silencing of circRNAs could be used as adjuvant for different therapies aimed at activating antitumor immune responses. As a future direction, novel investigations may be proposed to test in pre-clinical and clinical settings whether antisense oligonucleotides (ASOs) can be used to target one or more specific pro-tumorigenic circRNAs. Additionally, because virtually all tissues express circRNAs, other tumors in addition to sarcoma could potentially respond to circRNA targeting to stimulate interferon and inflammatory pathways.

Mechanistically, we showed that circRNAs repress pro-inflammatory factors by blocking the RNA sensor RIG-I^47^, while PKR and MDA5 are only marginally involved. However, it is still possible that additional RNA sensors such as toll-like receptors (TLR) and nucleic acid binding proteins, including PACT, NF90/110, and ADAR1p150, also play a role in this process. These elements were previously reported interacting with circRNAs by binding and competition screening assays in vitro^10^. Nevertheless, such binding still needs to be demonstrated in vivo. How might circRNAs bind and repress RIG-I activation? A recent publication showed that circRNAs can form one or multiple locally double-stranded RNA structures, making them amenable to interacting with cytosolic RNA sensors, including RIG-I^10^. Although the current study did not investigate the circRNAs’ structures, we may speculate that dsRNA secondary structures could allow circCsnk1g3 and circAnkib1 to bind one or multiple RIG-I molecules and repress the RIG-I-mediated pathways that drive the expression of interferons and inflammatory elements. Interestingly, both circRNAs were present at a copy number per cell that would in principle allow for a near-equimolar interaction with RIG-I.

Direct RNA/protein binding may not be the only mechanism underlying the circRNAs’ ability to modulate RIG-I function. Even though RIG-I was required for the two circRNAs to exert their immunomodulatory effects, their mutual enrichment in pulldowns was a modest 2- to 4-folds. Indeed, it has been demonstrated that circRNAs’ double-stranded regions are shorter than the lengths typical of a viral RNA^10^, which could explain the relatively weaker affinity between an RNA-binding protein and circRNAs. Interestingly, it has been suggested that the length of the binding region may determine whether the protein’s function is activated or blocked^10^. Finally, additional, binding-independent mechanisms may also contribute to the functional interaction. Further studies are thus required to reveal the physical interaction that underlies circCsnk1g3 and circAnkib1’s dependence upon RIG-I for their function in tumor cells.

These data open the possibility that many more circRNAs in addition to those described here could regulate inflammatory pathways and TME composition. Future investigations would be necessary to characterize the structures of functional circRNAs, and the stoichiometric relationship between the circRNAs, their putative dsRNA structures, and their protein binding partners. Such investigations will ultimately also assess the possibility that one circRNA could simultaneously sequester and regulate multiple binding proteins. Finally, although not investigated in the current study, the N6-adenosine methylation status of the circRNAs should also be investigated as part of the circRNAs capacity to activate RIG-I pathways. In this regard, it has been reported that methylation residues on endogenous circRNAs repress the activation of RIG-I pathways^30^. It would be essential to define the methylation status of the inflammatory circRNAs, including circCsnk1g3 and circAnkib1, and compare them to the circRNAs that are not capable of triggering pro-inflammatory signals. These analyses could potentially anticipate which circRNAs in each tumor type represent the most promising targets to enhance antitumor immunity, perhaps in combination with immunotherapies, DNA-demethylating agents, or mainstay treatments.

## Methods

### Mouse and human cell lines

Female, 8–10 wk old, C57Bl/6 J wild-type (strain #000664), C57Bl/6 J p53^KO^ (#002101), and athymic nude NU/J mice (#002019) were purchased from The Jackson Laboratory. Mice were housed at ambient room temperature (74 ± 2 °F) with humidity of 30–70% and a light/dark cycle of 14 h/10 h. Tumor burden did not exceed 1.5 cm^3^. Maximal tumor burden and all other aspects of animal experiments were performed in accordance with the guidelines of Cedars-Sinai Medical Center Institutional Animal Care and Use Committee. The human 293 T cells line for viral preparation was purchased from ATCC. 293 T were grown in DMEM, supplemented with glutamine, 10% fetal bovine serum (Gibco), 100 IU/ml penicillin, and 100 μg/ml streptomycin (Gibco). Cells were cultured in an incubator at 37 °C and 5% CO_2_.

### Mouse mesenchymal stromal cells isolation, maintenance, and in vivo tumorigenesis

Subcutaneous sarcomas were generated as previously described^24^. Briefly, long bones were collected from p53^KO^ mice, crushed, and digested with collagenase II (1 mg/ml) for 1 hour at 37 °C on a shaker. Recovered cells were stained and FACS-sorted as CD45^−^CD31^−^Ter119^−^Sca1^+^PDGFRα^+^ and cultured using complete MesenCult medium (STEMCELL Technologies). MSCs were maintained in a humidified chamber with 5% CO_2_ and 1% O_2_, with half of medium changed every 3 days. After 7 days in culture at 1% O_2,_ cells formed visible CFU-F colonies; after this point cells were periodically split at 80% confluency. To generate sarcoma cells, mesenchymal cells were transduced for the stable overexpression of *Ccne1* (see below for plasmid generation). The stable cells were assessed by RTqPCR and/or western blot, expanded in vitro and then used for in vivo tumorigenesis assays. Experiments measuring in vivo tumorigenesis (subcutaneous tumors) were carried out following the protocol previously described^24^. Briefly, 3D scaffolds (5 mm × 2 mm) made with reticulated polycarbonate polyurethane urea matrix (CS1-0502-25, DSM Biomedical/Biomerix) were seeded with MSCs at a concentration of 1 × 10^5^ cells/scaffold. Cells were allowed to adhere to the scaffolds for a minimum of 6 hours. Scaffolds were then implanted subcutaneously into mouse flanks, and tumors were harvested 3 weeks after implantation. To seed sarcomas in the lung, 2 × 10^5^ p53^KO^Ccne1^+^ sarcoma cells were injected intravenously in the mouse tail. Pulmonary sarcoma macroscopic foci were visible starting at 20 days post-injection. After isolation from primary recipient mice, sarcoma cells were cultured in DMEM, supplemented with glutamine, 10% fetal bovine serum, 100 IU/ml penicillin, and 100 μg/ml streptomycin (Gibco), and maintained at 37 °C and 5% CO_2_.

### RNA-sequencing of human and mouse sarcoma samples

Human undifferentiated sarcoma primary samples were collected, cryopreserved, and provided by the bio-repository of the Mount Sinai School of Medicine, NY. Slides from these specimens were reviewed by expert pathologists to verify histology, and a tissue cut was taken from each sample for RNA isolation. After homogenization, RNA was isolated with TRIzol (Invitrogen) and purified by RNA isolation kit (Invitrogen), according to the manufacturer’s instructions. Mouse tumors were surgically resected, the tissue was homogenized, and RNA was isolated and purified in a similar manner. For both mouse and human primary sarcoma samples, ribosomal RNAs were depleted from the total RNA before sequencing using the Ribo-Zero rRNA Removal kit (Epicentre). The TruSeq Stranded Total RNA library Prep kit (Illumina) was used to generate RNA-seq libraries from the primary sarcoma samples. Ribosomal RNA depletion, library preparation, sequencing, and bioinformatic analysis of the circRNAs were performed at the Center for Cancer Computational Biology at the Dana-Farber Cancer Institute. Circular RNA analysis was performed using KNIFE (v1.3)^48^. Only 117 circRNA-generating genes, detected with more than 1 read spanning the backsplice junction, were shared between multiple human samples (≥5 patients). 304 circRNAs were identified in mouse UPS with more than 1 read spanning the backsplice junction. Library preparation and mRNA-sequencing of the mouse sarcoma cells silenced for the expression of circCsnk1g3, circAnkib1, LinCsnk1g3, and LinAnkib1 was conducted at the Cedars-Sinai Center for Bioinformatics and Functional Genomics. Raw sequencing data were demultiplexed and converted to fastq format by using bcl2fastq v2.20 (Illumina, San Diego, California). Then reads were aligned to the GRCm38 reference genome (http://www.gencodegenes.org) using STAR (version 2.6.1)^49^ with default parameters. Gene expression was quantified by RSEM (version 1.2.28)^50^ to generate a raw count expression matrix with gene identities as rows and samples as columns. Differential expression analysis was conducted in edgeR (v3.30), and gene lists pre-ranked by *p* value were used as input to Gene Set Enrichment Analysis as implemented in the *clusterProfiler* R package (v3.16)^51,52^.

### Generation of retrovirus, lentivirus, knockdown, knockout, and overexpressing cells

The retroviral vector pCMMP-MCS-IRES-mRFP (Addgene #36972) was used for the overexpression of the *Ccne1* gene. The gene was amplified from the cDNA of mouse mesenchymal cells and cloned into the retroviral vector by using the Gibson Assembly kit (New England BioLabs). The expression of the transgene was verified by RTqPCR. The shRNAs, including shCircRNAs, were cloned into the pLKO.1 lentiviral vector (Addgene #10879), following Addgene protocols. The shRNA sequences were designed according to the following program provided by the Broad Institute: https://portals.broadinstitute.org/gpp/public/seq/search. The sequences are provided in Supplementary Data 3. CRISPR sgRNAs were cloned in a Cas9-GFP expressing lentiviral vector (Addgene #82416). Cas9-GFP^+^ cells were FACS-sorted and tested by RTqPCR before experiments. The CRISPR sgRNA sequences were designed according to the following program provided by the Broad Institute: https://portals.broadinstitute.org/gpp/public/analysis-tools/sgrna-design.

All the viral particles were produced in 293 T cells, which were co-transfected with the specific viral vector and packaging-expressing plasmids: pECO for the retroviral vectors, and VSV-G, REV, and d8.74 for the lentiviral vectors. Transfection of the cells was performed by using Lipofectamine 3000 diluted in Opti-MEM, according to manufacturer instructions. Transfection medium was changed 8 hours after transfection, and the lentiviral particles were collected 24 and 48 hours after transfection. Viral supernatant was used with polybrene (10 μg/ml) to infect the sarcoma cells, which were seeded at a confluence of 50% the day prior to transduction. Sarcoma cells were incubated overnight with the viral supernatant, washed with PBS, and then supplemented with a complete medium. Antibiotic selection (puromycin 2 μg/ml) was performed at least 72 hours post-infection.

### RNA extraction, PCR and RTqPCR

Total RNA was extracted using TRIzol (Invitrogen/Life Technologies) according to the manufacturer’s instructions. To enrich circRNA isoforms, RNAse-R treatment was carried out for 15 minutes at 37 °C using 2U RNAse-R (Epicentre) per 1 μg of RNA. Treated RNA was directly reverse transcribed using the High-Capacity cDNA Reverse Transcription Kit (Applied Biosystems/Life Technologies) according to manufacturer instructions. 5–10 ng of RNA were used for each PCR reaction; *Tbp* (TATA-box binding protein) or *ACTB* (β actin) was used as housekeeping gene for the RTqPCR experiments. Quantitative PCRs were carried out using SYBR Green PCR Master Mix and QuantStudio 3 Real-Time PCR system (Applied Biosystems/Life Technologies). Primer sequences are provided in Supplementary Data 3. In selected experiments, we used convergent primers to detect the linear transcripts and divergent primers to identify the circRNAs. For the RTqPCR assays aimed to detect interferon genes upon circRNA-KD, 6 × 10^4^ cells were seeded, starved overnight in medium without serum, and then re-stimulated for at least 48 hours before RNA extraction. In indicated experiments, cells were treated with abemaciclib (CDK4/6 inhibitor, 250 mM for 4 days) prior to RNA extraction and quantification. For the pulldown experiments, RTqPCR data are shown either as percent of input, or relative to IgG control and normalized on *Tbp*. RTqPCR data are plotted using GraphPad Prism v8.

CircRNA copy numbers were measured by using a standard curve. For each circRNA, a standard curve was generated from serial dilutions of purified PCR product of the backsplice junction, and copy numbers were calculated based on molecular weight of the PCR product. RNA was extracted from a known number of mouse UPS cells and reversed transcribed. 2,000-cell aliquots of cDNA were used to detect circRNAs by qPCR.

### CircFISH

The circRNAs were imaged using a modification of single molecule FISH assay as demonstrated previously^53^. Briefly, a set of 40 probes each 20 nt long was designed to bind specifically to the exons that become part of both the circRNA and the linear isoform, and another set was designed against the exons exclusively present in linear RNA. Each probe was ordered with a 3’ amino modification. The probes for each set were pooled and labeled en masse with different fluorophores and purified using HPLC. The circ+linRNA probe set was labeled with Cy5 and linear-only probe set was labeled with Texas Red, such that any red or yellow signal corresponded to detection of a linear transcript, while green signal corresponded to detection of a circRNA. The cells were grown on glass coverslips, fixed, permeabilized and hybridized with probes overnight. The coverslips were washed, mounted, and imaged using Nikon TiE inverted fluorescence microscope equipped with Pixis1024B camera in 100X oil objective. For simultaneous imaging of proteins, immunofluorescence staining was performed after the overnight hybridization with probes, and then the cells were imaged^54^. Images were acquired using Metamorph and processed using custom-written programs in MATLAB (Mathworks Inc)^55^.

### Immunofluorescence

For dsRNA immunofluorescence, mouse sarcoma cells were fixed on a staining chamber with 4% PFA for 10 min, washed with PBS, and permeabilized with PBS, Triton X-100 0.2% for 10 min. Blocking before antibodies was performed in PBS, Triton X-100 0.2 and 10% FBS for 30 min. Primary anti-dsRNA antibody (J2, Jena Bioscience) was incubated overnight in blocking buffer, followed by incubation with Alexa Fluor 488-conjugated goat anti-mouse IgG (H + L) secondary antibody (A32723, Invitrogen/Thermo Fisher).

For CD3 and FoxP3 immunofluorescence, tumor tissues were collected from mice, and embedded in paraffin after formalin fixation at 4 °C. Tissue sections were heated at 65 °C for 60–90 min, deparaffinized in xylene, rehydrated in an ethanol gradient, and submerged in Tris EDTA (pH 9.0) and heated for 20 min to unmask antigens. Following blocking with a universal protein blocking reagent, serial sections were incubated with anti-CD3 primary antibody (clone SP7, 1:200 dilution) (#NB-600-1441, Novus Biologicals) for 2 hours at room temperature, washed with TBST buffer three times, and incubated with secondary anti-rabbit IgG H&L HRP-conjugated antibody (ab214880, Abcam, prediluted). Staining signal was amplified using Opal reagents for tyramide signal amplification (FP1487001KT and FP488001KT, Akoya Biosciences). For FoxP3 co-staining, sections underwent a second round of processing, but using citrate buffer for antigen retrieval and anti-FoxP3 Rabbit mAb (clone D6O8R, 1:100 dilution) (#12653, Cell Signaling) for primary antibody staining. Nuclei were counterstained with DAPI.

### Flow cytometry

Cells were analyzed using the LSR II (BD, Pharmingen) and sorted using FACSAria III (BD, Pharmingen), using FACSDiva software (BD, v8). Analysis was performed with FlowJo (v10). Samples were stained with the following antibodies at 1 ul antibody per 100ul cell suspension: anti-CD45 FITC, anti-CD31 FITC, anti-Ter119 FITC, anti-Sca1 Pacific Blue, anti-PDGFRα PE, anti-CD3 APC, anti-CD8 FITC, anti-CD45 Pacific Blue, anti-CD4 PE (all purchased from Biolegend). Tumors were enzymatically digested to single-cell suspension and filtered twice through 70 μm filters. Red blood cells were lysed with ACK solution (Gibco), washed twice with PBS, and then stained with the fluorophore-conjugated antibodies for 15 minutes at room temperature. The excess of unbound antibodies was washed out before acquisition in flow cytometry. Intracellular staining was performed to detect dsRNAs. Briefly, cells were fixed and permeabilized by using Cyto-Fast fix/perm buffers (Biolegend), following vendor’s protocol. After performing the staining for the surface markers, J2 antibody (Jena Bioscience, 2ug total) was added to the cells and incubated for 1 hour at room temperature, followed by incubation for 20 minutes with Alexa Fluor 488-conjugated goat anti-mouse IgG (H + L) secondary antibody (A32723, Invitrogen/Thermo Fisher).

### Western blot and RNA Immunoprecipitation

Protein lysates were prepared with ice-cold RIPA buffer (Boston Bio Products) supplemented with protease and phosphatase inhibitors (Roche). Protein lysates were separated using NuPAGE 4 to 12% Bis-Tris precast electrophoresis gels (NP0335BOX, Invitrogen) and transferred to a nitrocellulose membrane. After blocking the membrane with 5% milk in PBST (PBS with 0.1% Tween20, Sigma), the membranes were incubated overnight at 4 °C with anti-MDA5 (#5321, Cell Signaling), anti-RIG-I (#3743, Cell Signaling), anti-β actin (A300-485A, Bethyl), anti-TBK (#3504, Cell Signaling), anti-pTBK (#5483, Cell Signaling), or anti-IRF1 (#8478, Cell Signaling) primary antibodies, diluted 1:1000 in PBST with 5% BSA. The blots were washed 3 times in PBST, incubated with secondary anti-rabbit HRP antibody (31460, Invitrogen/Thermo Fisher) diluted 1:2000 in PBST with 5% milk, and washed 3 more times. Finally, the membranes were incubated with SuperSignal Femto or Pierce ECL substrate (Thermo Scientific) for 1 minute and exposed for signal detection with the iBright imaging system (Invitrogen). Images were quantified in ImageJ (NIH, v1.52k).

For RNA-immunoprecipitation (RIP) experiments, cells were lysed with Cell Lysis Buffer (Cell Signaling Technology #9803), supplemented with complete protease inhibitor cocktail (Roche) and RNAse inhibitors (Thermo Fisher). Lysate was first pre-cleared with Protein-G magnetic beads (Invitrogen), 1 h at 4 °C in rotation. The pre-clearing was repeated two times. After pre-clearing, the lysate was incubated overnight with rabbit IgG as control or with anti-RIG-I (D14G6) antibody (Cell Signaling #3743), diluted 1:100. Protein-G magnetic beads (Invitrogen) were then used to isolate the RIG-I/RNA complexes, 4 hours in rotation at 4 °C. Samples were then partially used for western blot to control the efficiency of the RIG-I pulldown, and partially resuspended in TRIzol (Invitrogen) for RNA extraction. For western blot, proteins were eluted from beads by boiling them with SDS-sample buffer (Boston BioProducts) for 10 minutes. Western blot was then performed to detect RIG-I with the antibody indicated above.

### CRISPR-assisted RNA-protein interaction detection (CARPID)

BASU-dCasRx expressing sarcoma cells were generated by transduction with the BASU-dCasRx plasmid (Addgene 153209), and sorted for GFP expression. The expression of the CasRx was evaluated by RTqPCR. We designed sgRNAs targeting the backsplice junction of circCsnk1g3 and circAnkib1. The sgRNAs were cloned into the pLentiRNAGuide plasmind (Addgene 138150), and added to the cells expressing the BASU-dCasRx. Transduced cells were selected by puromycin treatment. Cells at confluency in one 15-cm plate were treated with 200 μM biotin for 15 min at 37 °C, washed three times with ice-cold PBS (Thermo Fisher) and harvested by scraping in 2 ml of ice-cold PBS. Cells were spun down at 1200 r.p.m. for 5 minutes and lysed with 1 ml lysis buffer (Cell Signaling Technology #9803) supplemented with fresh protease inhibitors (Roche) at 4 °C for 10 minutes with end-over-end rotation. Then, lysate was spun down at 15,000 r.p.m. for 10 minutes at 4 °C. The supernatant was quantified and normalized for protein concentration, which was sampled for input. Biotinylated proteins were enriched with MyOne T1 streptavidin beads (Thermo Fisher) after 2 h of incubation at 4 °C with end-over-end rotation, and three washes were performed by end-over-end rotation with 1 ml ice-cold lysis buffer for 5 minutes. Proteins were eluted from the beads into elution SDS-sample buffer (Boston BioProducts) by incubation for 10 min at 95 °C.

### Reverse phase protein array (RPPA)

Cells (5 × 10^6^) were seeded in 10 cm^2^ plates and grown overnight. Cells were then washed with PBS, detached from the plates with trypsin digestion, and washed with cold PBS three times. The cell pellet was frozen in liquid nitrogen and stored at −80 °C. The RPPA analysis was performed at the Functional Proteomics RPPA Core Facility at the MD Anderson Cancer Center, where cells were lysed in 1% Triton X‐100, 50 mM HEPES, pH 7.4, 150 mM NaCl, 1.5 mM MgCl_2_, 1 mM EGTA, 100 mM NaF, 10 mM Na pyrophosphate, 1 mM Na3VO4, 10% glycerol, containing freshly added protease and phosphatase inhibitors from Roche Applied Science Cat. # 05056489001 and 04906837001, respectively. Cell lysate samples were serially diluted and arrayed on nitrocellulose-coated slides to produce sample spots, which were then probed with antibodies by a tyramide-based signal amplification approach and visualized by DAB colorimetric reaction to produce stained slides. Stained slides were scanned on a Huron TissueScope scanner to produce 16-bit tiff images. Sample spots in tiff images were identified and their densities were quantified by Array-Pro Analyzer. Relative protein levels for each sample were determined by interpolating each dilution curve produced from the densities of the 5-dilution sample spots using a “standard curve” (SuperCurve) for each slide (antibody). All relative protein level data points were normalized for protein loading.

### Single-cell RNA sequencing

For each tumor, sarcoma cells were seeded with cell culture medium into a 5 mm diameter × 2 mm thick Biomerix Biomaterial (polycarbonate polyurethane urea) scaffold (CS1-0502-25, DSM Biomedical/Biomerix), and incubated overnight. The scaffolds were implanted subcutaneously into sex- and age-matched mice via a small incision on the shoulder. Resulting tumors were dissected after 3 weeks. Tumors were minced and then enzymatically and mechanically digested using the *37C_m_TDK_2* protocol on the Miltenyi gentleMACS with Tumor Dissociation Kit for Mouse (Miltenyi Biotec, Auburn, CA). Cell suspensions were washed in DPBS with 0.04% BSA and filtered through 70 μm strainers (Bioland Scientific LLC, Paramount, CA). Red blood cells were lysed with ACK buffer. To enrich the cell suspension for immune cells, samples were stained with anti-CD45 antibody-conjugated to magnetic particles using the CD45 EasySep selection kit (Stemcell Technologies, Vancouver, BC), and separated into positive and negative fractions using a magnet. CD45-selected cell suspensions were each tagged with a unique TotalSeq-A Hashtag reagent (BioLegend, San Diego, CA)—an antibody-conjugated oligo barcode. Final cell suspensions were washed three times in PBS, filtered through 40 μm Bel-Art FlowMi strainers (Bel-Art/SP Scienceware, Wayne, NJ), counted, and pooled into a single sample at a concentration of 1000 cells/μL. The cell suspension was loaded into the Chromium Controller (10X Genomics, Pleasanton, CA) for cell lysing and mRNA capture, with the Chromium Single-Cell 3’ Reagent Kit (v3 chemistry). Sequencing libraries were prepared using the same kit according to standard protocol and sequenced by the Cedars-Sinai Genomics Core on the NovaSeq (Illumina, San Diego, CA) at 400 M reads. Sequencing data were demultiplexed, and then converted to read counts using the Cell Ranger (10X Genomics, v6) pipeline.

The Seurat R package (v4)^56^, RStudio (v1.3), and R (v4.0) was used for all subsequent analysis. *HTODemux* was used to call sample hashes for each cell, and to exclude likely doublets bearing more than one hashtag. Low-quality cells (with < 200 genes detected or with mitochondrial gene counts >10%) were removed. Gene expression values were normalized and scaled with *SCTransform*, aligned across conditions with *FindIntegrationAnchors* and *IntegrateData*, and reduced to principal components with *RunPCA*. Cell nearest neighbors were calculated using the top 30 PCs, and cell clusters were determined by the Louvain algorithm with the resolution parameter at 0.6.

### Reporting summary

Further information on research design is available in the Nature Portfolio Reporting Summary linked to this article.

## Supplementary information

Supplementary Information

Description of Additional Supplementary Files

Supplementary Data 1

Supplementary Data 2

Supplementary Data 3

Supplementary Data 4

Supplementary Data 5

Supplementary Data 6

Reporting Summary

## Data Availability

RNA-seq and scRNA-seq data have been deposited in the NCBI Gene Expression Omnibus under accession GSE163202. All other original data supporting the findings of this study are included in the Source Data files and Supplementary Information. The MiOncoCirc database is publicly accessible at https://mioncocirc.github.io/. Source data are provided with this paper.

## Source data

Source Data
