# Supplementary material for: circCsnk1g3- and circAnkib1-regulated interferon responses in sarcoma promote tumorigenesis by shaping the immune microenvironment
Source: Nat Commun. 2022 Nov 25;13:7243. doi: 10.1038/s41467-022-34872-8 (PMC9700836; doi:10.1038/s41467-022-34872-8)
Supplement: Supplementary file 1 — Supplementary Information [file 41467_2022_34872_MOESM1_ESM.pdf]

Ko, Piras et al, supplementary Figure 1

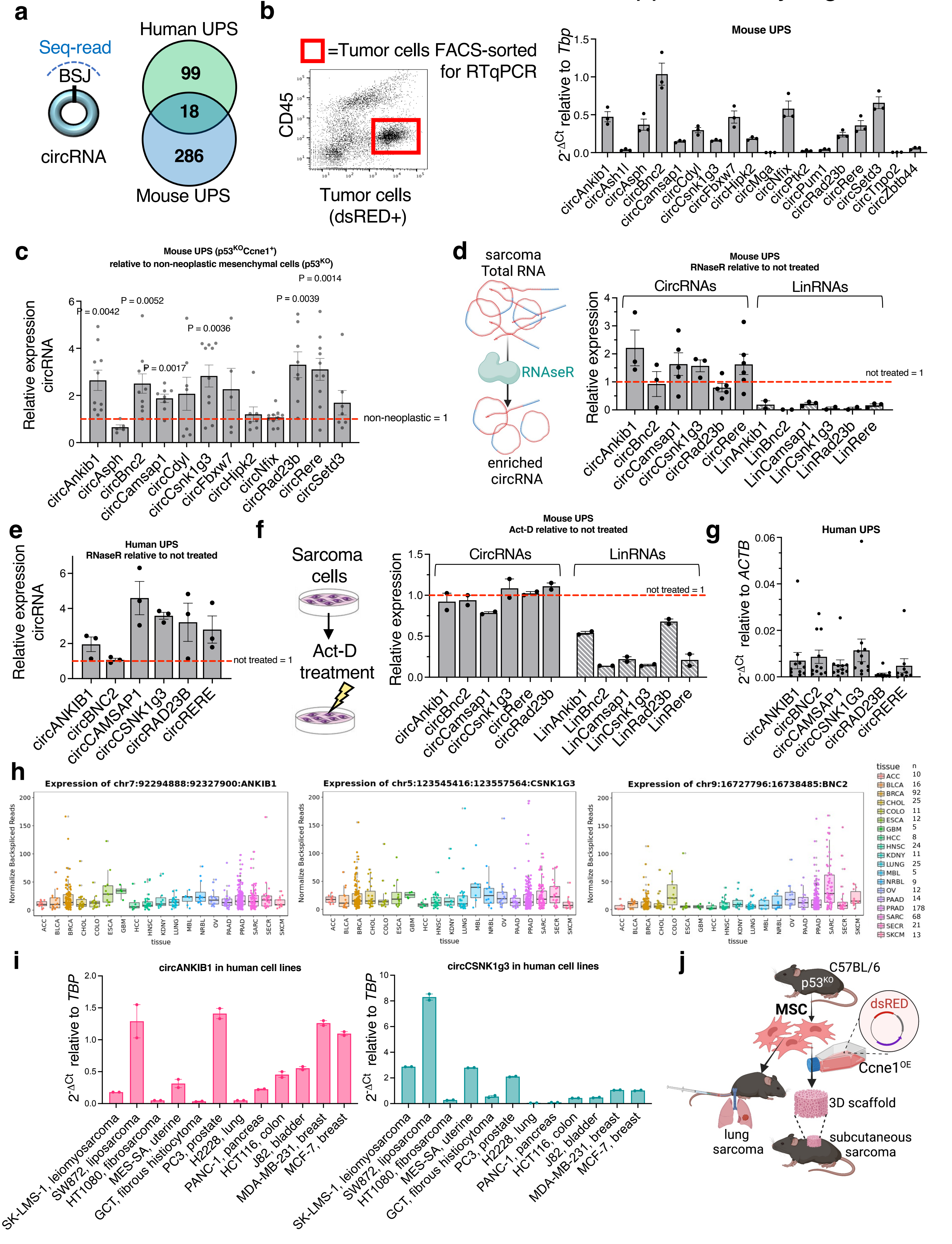

Supplementary Fig. 1 Expression of circRNAs in undifferentiated pleiomorphic sarcoma.
(**a**) Left: schematic of RNA-sequencing reads spanning the back-splice junction, used to identify circRNAs. Right: schematic representation of the unique and shared circRNAs identified in human and mouse primary UPS samples, analyzed by RNA-sequencing. BSJ: backsplice junction. (**b**) Left: representative gating strategy to FACS-sort sarcoma cells (dsRED+) from subcutaneous mouse tumor. Right: RTqPCR validation of the expression in ex-vivo mouse UPS cells of the 18 circRNAs detected by both human and mouse RNAseq. Data are reported as mean $2^{-\Delta Ct}$ ± s.e.m. (normalized on *Tbp*), and dots represent n=3 independent biological replicates. (**c**) RT-qPCR analysis of candidate circRNAs' expression in mouse UPS cells ($p53^{KO}Ccne1^{+}$), relative to non-malignant ($p53^{KO}$) cells (dotted line). Data are reported as mean fold-change ± s.e.m. Dots represent n ≥ 4 independent biological replicates. *P* values: two-tailed one sample t-test. (**d**) RTqPCR analysis of the expression of candidate circRNAs (left) and their linear counterparts (right) in mouse sarcoma cells treated with RNase R, relative to not treated (dotted line). Data are reported as mean fold-change in RNAse R-treated vs untreated RNA, ± s.e.m. Dots represent independent biological replicates. (**e**) RTqPCR expression of the candidate circRNAs in human primary sarcoma samples (n=3 samples) after treatment with RNAse R, relative to not treated (dotted line). Data are reported as mean fold-change in RNAse R-treated vs untreated RNA, ± s.e.m. Dots represent independent samples. (**f**) Left: Schematic of the experiment to measure half-life of linear and circular transcripts following transcription blockade with Act-D. Right: RT-qPCR analysis of candidate circRNAs (left) and linear counterparts (right) in the sarcoma cells 20 hours after treatment with Act-D, relative to not treated (dotted line). Technical replicates from one experiment are shown. (**g**) RTqPCR validation of the conserved, tumor-enriched circRNAs in human primary sarcoma samples. Data are reported as mean $2^{-\Delta Ct}$ ± s.e.m. (normalized on *ACTB*) and dots represent independent samples. (**h**) circANKIB1, circCSNK1G3, and circBNC2 expression queried in MiOncoCirc. Data are presented as median, 25th & 75th percentiles (box) ± 1.5 x IQR (whiskers). Dots represent individual tumor samples. (**i**) Expression of circANKIB1 (left) and circCSNK1G3 (right) in human tumor cell lines of both mesenchymal and epithelial cancers. Technical replicates from one experiment are shown. (**j**) Schematic of mouse models used to generate subcutaneous and lung sarcoma. Source data are provided as Source Data File.

Ko, Piras et al, supplementary Figure 2

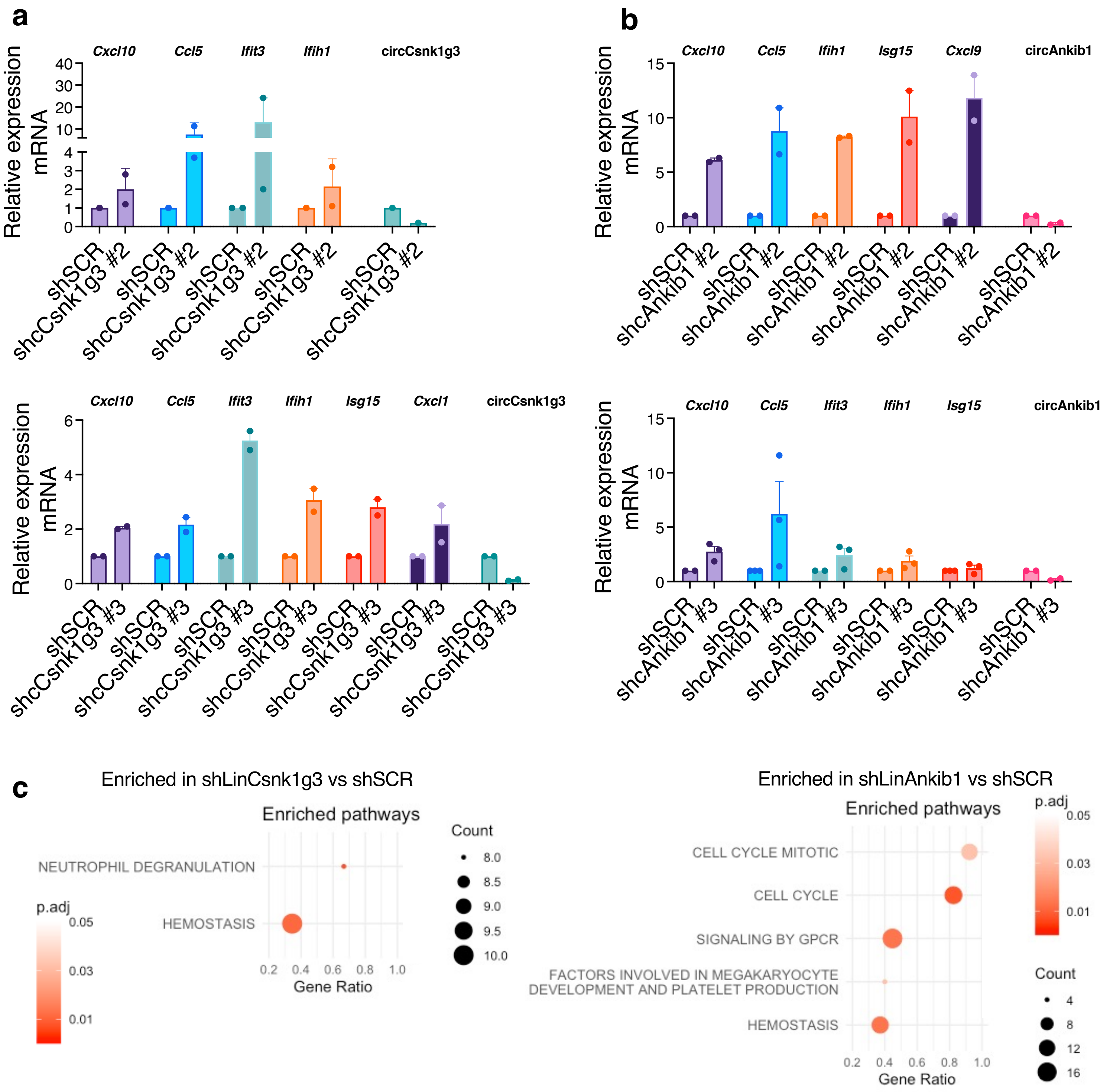

Supplementary Fig. 2 circRNAs regulate the expression of interferon signals and pro-inflammatory cytokines in tumor cells. (**a**) RTqPCR showing the differential expression of interferon-related genes after silencing of circCsnk1g3, using two alternative shcircRNA designs. Data are reported as mean fold-change ± s.e.m. Dots represent biological replicates from n=2 independent experiments for the interferon-related genes, and 2 technical replicates for confirming circCsnk1g3 knockdown (rightmost bar). (**b**) RTqPCR showing the differential expression of interferon-related genes after silencing of circAnkib1, using two alternative shcircRNA designs. Data are reported as mean fold-change ± s.e.m. Dots represent technical replicates of n=2 independent experiments with similar trend (top) or n=3 (bottom) biological independent experiments. Technical replicates are shown for confirming circAnkib1 knockdown (rightmost bar). (**c**) Differential expression analysis of the top pathways enriched upon knockdown of linear *Csnk1g3* (left) and linear *Ankib1* (right). Pathways are colored by Benjamini-Hochberg adjusted P value. Source data are provided as Source Data File.

Ko, Piras et al, supplementary Figure 3

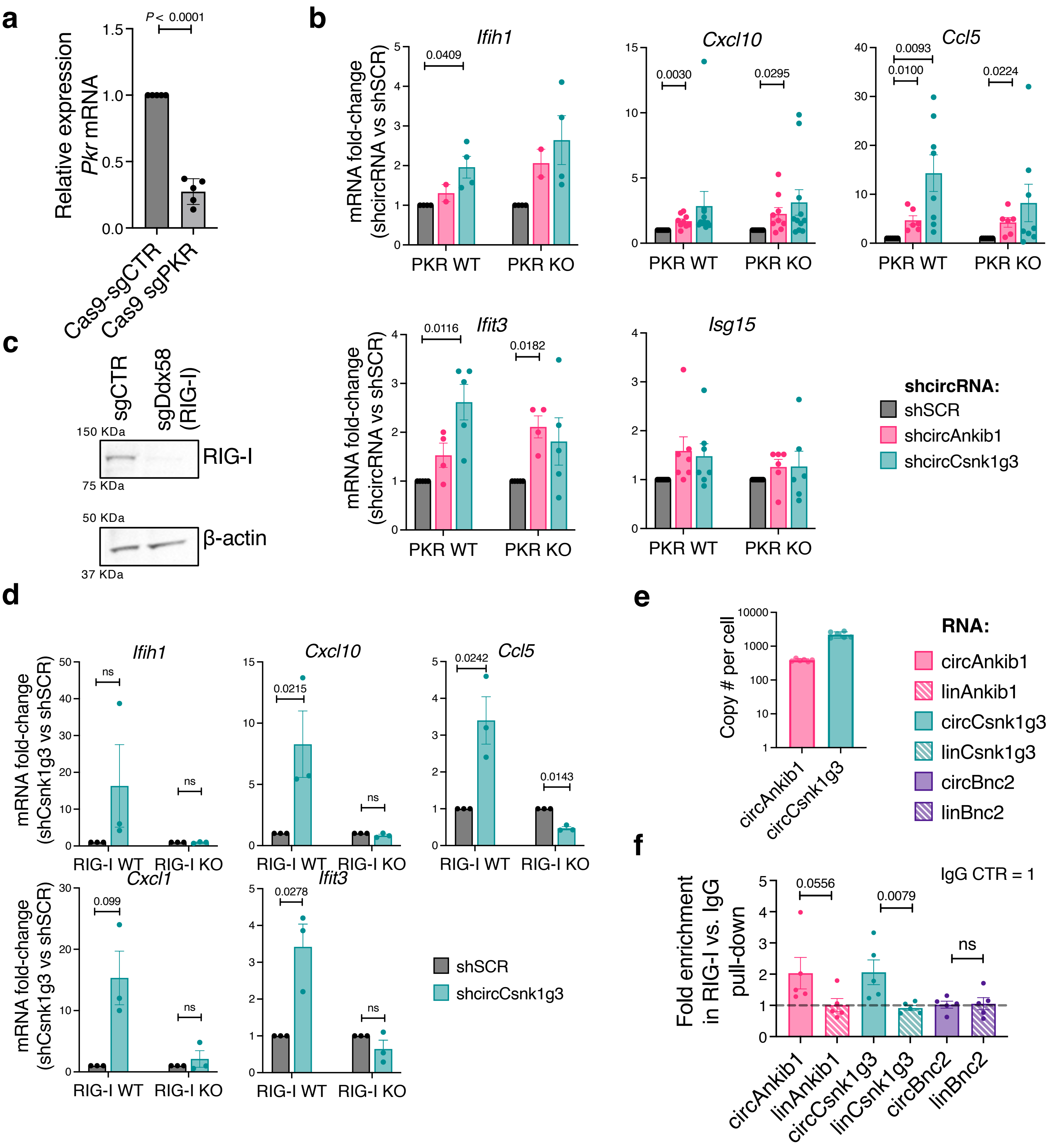

Supplementary Fig. 3 circRNAs regulate the expression of interferon genes through RIG-I.
(**a**) Validation of reduced *Pkr* expression in the PKR-KO cells. Dots represent n=5 biological replicates. (**b**) RTqPCR quantification of interferon-related genes upon knockdown of circCsnk1g3 or circAnkib1, in Cas9-expressing cells of either control (PKR WT) or knocked out for the expression of PKR (PKR KO). Dots represent biological replicates. *P* values: two-tailed one sample t test. (**c**) Validation of protein knockout of RIG-I (n=1). (**d**) RTqPCR quantification of interferon-related genes upon knockdown of circCsnk1g3, in Cas9-expressing cells of either control (RIG-I WT, n=3 biological replicates) or knocked out for the expression of RIG-I (RIG-I KO, n=3 biological replicates). *P* values: two-tailed one sample t test. (**e**) Estimated copy numbers per cell of circAnkib1 and circCsnk1g3, determined in RTqPCR by measuring circRNAs by RTqPCR in known numbers of cells. Dots represent n=6 biologically independent samples. Data are reported as mean with 95% CI. (**f**) Detection of circRNAs, as well as their linear counterparts, in RNA immunoprecipitation by anti-RIG-I antibody or by IgG control. Data are represented as ratios (abundance in the RIG-I pull-down compared to IgG pull-down), and normalized on internal housekeeping gene (*Tbp*). Dots represent n=5 independent repetitions. *P* values: unpaired Student's t test. For all figures, data are reported as mean ± s.e.m. Source data are provided as Source Data File.

Ko, Piras et al, supplementary Figure 4

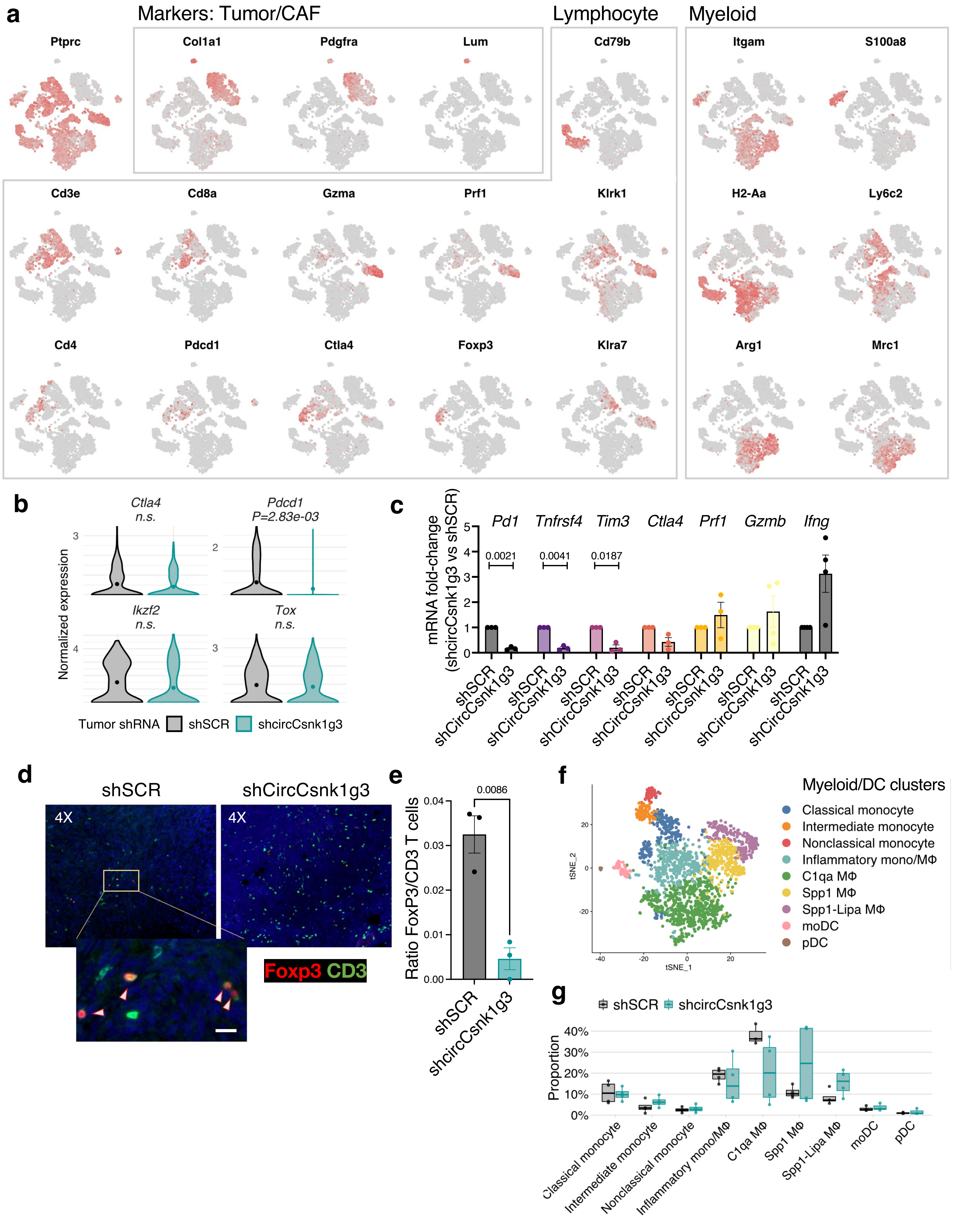

Supplementary Fig. 4 Targeting circRNAs in tumor cells re-shapes the tumor microenvironment. (**a**) tSNE plot overlaid with normalized expression of marker genes used to distinguish the main tumor and TME populations captured in single-cell RNA-sequencing. (**b**) Expression of selected genes in CD8$^+$ T cells from shSCR (n=4) and shcircCsnk1g3 (n=4) tumors, analyzed by single-cell RNA-sequencing. n=428 and n=375 CD8$^+$ T cells were derived from shSCR and shcircCsnk1g3 tumors, respectively. Mean expression values are plotted as dots and *P* values derived by Wilcoxon rank sum test with Bonferroni correction. (**c**) RTqPCR analysis of FACS-sorted CD8$^+$ T cells from subcutaneous sarcomas of control (shSCR) or in which the expression of circCsnk1g3 was silenced (shCircCsnk1g3). Data are reported as mean fold-change ± s.e.m. and dots represent individual mice. *P* values: two-tailed one sample t test. (**d**) FoxP3 and CD3 staining in representative sections of shcircCsnk1g3 (n=3) and shSCR tumors (n=3). (**e**) FoxP3$^+$ Treg cells as fraction of CD3$^+$ T cells in shcircCsnk1g3 (n=3) and shSCR (n=3) tumor sections. Data are reported as mean ± s.e.m. and dots represent independent sections. *P* value: Welch's t test. (**f**) tSNE plot of myeloid and dendritic cell subclusters. (**g**) Comparative proportions of myeloid and dendritic cell types in scRNA-seq of shcircCsnk1g3 and shSCR tumors. Data are reported as median, IQR (box) and 1.5 x IQR (whiskers). Dots represent individual mice. Source data are provided as Source Data File.
