# Supplementary material for: circCsnk1g3- and circAnkib1-regulated interferon responses in sarcoma promote tumorigenesis by shaping the immune microenvironment
Source: Nat Commun. 2022 Nov 25;13:7243. doi: 10.1038/s41467-022-34872-8 (PMC9700836; doi:10.1038/s41467-022-34872-8)
Supplement: Supplementary file 2 — Description of Additional Supplementary Files [file 41467_2022_34872_MOESM2_ESM.pdf]

Description of Additional Supplementary Files

Supplementary Data 1.
Circular RNAs detected in human UPS samples.

Supplementary Data 2.
Circular RNAs detected in mouse UPS samples.

Supplementary Data 3.
Oligonucleotide sequences used for shRNAs, sgRNAs, and qPCR primers.

Supplementary Data 4.
Markers of all high-level cell types in scRNAseq.

Supplementary Data 5.
Markers of lymphocytic cell types in scRNAseq.

Supplementary Data 6.
Markers of myeloid and dendritic cell types in scRNAseq.
