# Supplementary material for: circCsnk1g3- and circAnkib1-regulated interferon responses in sarcoma promote tumorigenesis by shaping the immune microenvironment
Source: Nat Commun. 2022 Nov 25;13:7243. doi: 10.1038/s41467-022-34872-8 (PMC9700836; doi:10.1038/s41467-022-34872-8)
Supplement: Supplementary file 9 — Reporting Summary [file 41467_2022_34872_MOESM9_ESM.pdf]

nature portfolio

Corresponding author(s): Jlenia Guarnerio

Last updated by author(s): Oct 26, 2022

Reporting Summary

Nature Portfolio wishes to improve the reproducibility of the work that we publish. This form provides structure for consistency and transparency in reporting. For further information on Nature Portfolio policies, see our Editorial Policies and the Editorial Policy Checklist.

Statistics

For all statistical analyses, confirm that the following items are present in the figure legend, table legend, main text, or Methods section.

n/a	Confirmed	
☐	☒	The exact sample size (*n*) for each experimental group/condition, given as a discrete number and unit of measurement
☐	☒	A statement on whether measurements were taken from distinct samples or whether the same sample was measured repeatedly
☐	☒	The statistical test(s) used AND whether they are one- or two-sided<br>*Only common tests should be described solely by name; describe more complex techniques in the Methods section.*
☐	☒	A description of all covariates tested
☐	☒	A description of any assumptions or corrections, such as tests of normality and adjustment for multiple comparisons
☐	☒	A full description of the statistical parameters including central tendency (e.g. means) or other basic estimates (e.g. regression coefficient) AND variation (e.g. standard deviation) or associated estimates of uncertainty (e.g. confidence intervals)
☐	☒	For null hypothesis testing, the test statistic (e.g. *F*, *t*, *r*) with confidence intervals, effect sizes, degrees of freedom and *P* value noted<br>*Give P values as exact values whenever suitable.*
☒	☐	For Bayesian analysis, information on the choice of priors and Markov chain Monte Carlo settings
☒	☐	For hierarchical and complex designs, identification of the appropriate level for tests and full reporting of outcomes
☒	☐	Estimates of effect sizes (e.g. Cohen's *d*, Pearson's *r*), indicating how they were calculated

Our web collection on statistics for biologists contains articles on many of the points above.

Software and code

Policy information about availability of computer code

Data collection	Flow Cytometry data were acquired using the BC FACSDiva software v8, and the BD LSR II instrument; cell sorting was performed with the BD FACSAria III instrument. CD45+ cells isolation for single cell RNA-sequencing was performed with magnetic beads recovered with the EasySep magnet. Western blot images were collected with the iBright imager (Thermo Fisher). RTqPCR data were generated with QuantStudio 3 (Applied Biosystems). Single-cell partitioning was performed by using a 10X Chromium controller. H&E for the tumor sections were acquired by using a bright field microscope (Evos XL Core, Thermo Fisher).
Data analysis	All graphs were generated using Graphpad Prism v8 or R v4.0. T-test P-value was calculated either with Graphpad Prism v8 or with Microsoft Excel. Western blot quantification was performed with ImageJ v1.52k. Flow Cytometry data were analyzed using FlowJo software v10. RNAseq and scRNAseq analyses used the following software: STAR v2.6.1, bcl2fastq v2.20 (Illumina), RSEM v 1.2.28, edgeR v3.30; GSEA/clusterProfiler v3.16, Cell Ranger v6 (10X Genomics), Seurat v4, R v4.0, RStudio v1.3, KNIFE v1.3.

For manuscripts utilizing custom algorithms or software that are central to the research but not yet described in published literature, software must be made available to editors and reviewers. We strongly encourage code deposition in a community repository (e.g. GitHub). See the Nature Portfolio guidelines for submitting code & software for further information.

Data

Policy information about availability of data

All manuscripts must include a data availability statement. This statement should provide the following information, where applicable:

- Accession codes, unique identifiers, or web links for publicly available datasets
- A description of any restrictions on data availability
- For clinical datasets or third party data, please ensure that the statement adheres to our policy

RNA sequencing and scRNA-seq data have been deposited as a Super Series at the NCBI Gene Expression Omnibus (GEO), under Accession Number GSE163202. Data analyzed in all figures of this paper are included in the Source Data file. Any other experimental data will be available from the corresponding author upon request. The MiOncoCirc database is publicly accessible at https://mioncocirc.github.io/.

Human research participants

Policy information about studies involving human research participants and Sex and Gender in Research.

Reporting on sex and gender	Not available
Population characteristics	Not available
Recruitment	Not available
Ethics oversight	De-identified human UPS samples were used, so no population data are available and IRB review was not required.

Note that full information on the approval of the study protocol must also be provided in the manuscript.

Field-specific reporting

Please select the one below that is the best fit for your research. If you are not sure, read the appropriate sections before making your selection.

☒ Life sciences ☐ Behavioural & social sciences ☐ Ecological, evolutionary & environmental sciences

For a reference copy of the document with all sections, see nature.com/documents/nr-reporting-summary-flat.pdf

Life sciences study design

All studies must disclose on these points even when the disclosure is negative.

Sample size	Sample size was calculated based on a pilot experiment to define the differential tumor size between control and experimental groups. Based on that, we calculated that data from 8 mice per group achieve 80% power to detect an effect size of 1.7 at the Bonferroni correct 0.025 level of significance using a two-sample t-test. The effect size if defined as the difference in mean tumor volumes divided by the common standard deviation.<br>All the independent replicates of the in vitro experiments are shown in the figures (single dots). For the mouse experiments, each mouse is shown as a dot in the presented charts.
Data exclusions	For circRNA knockdown experiments, if the expression of the circRNAs was not efficiently silenced (> 50% reduction compared to the controls, as measured by RTqPCR), data from these samples were excluded.
Replication	All studies, unless otherwise indicated, were confirmed at least three times with the exception of the Basu CasRx (twice) and the RPPA analysis that was performed one time. Data from the RPPA experiment were independently validated with complementary experiments, as reported in the manuscript. For in vivo experiments, multiple mice were used to ensure reproducibility.
Randomization	For in vivo experiments, animals were randomized to each experimental cohort.
Blinding	Animal experiments were not performed in a blinded manner, since no treatment experiments were performed. However, in some repetitions, tumor measurement and analysis was performed by operators blinded to the experimental groups. For the in vitro studies blinding was not relevant as all measures were quantified by standard cellular and biochemical assays. Key results were validated by 2 independent operators.

Reporting for specific materials, systems and methods

We require information from authors about some types of materials, experimental systems and methods used in many studies. Here, indicate whether each material, system or method listed is relevant to your study. If you are not sure if a list item applies to your research, read the appropriate section before selecting a response.

Materials & experimental systems

n/a	Involved in the study	
☐	☒	Antibodies
☐	☒	Eukaryotic cell lines
☒	☐	Palaeontology and archaeology
☐	☒	Animals and other organisms
☒	☐	Clinical data
☒	☐	Dual use research of concern

Methods

n/a	Involved in the study	
☒	☐	ChIP-seq
☐	☒	Flow cytometry
☒	☐	MRI-based neuroimaging

Antibodies

Antibodies used

Flow Cytometry antibodies:
anti-CD45 FITC (clone 30-F11, Biolegend #103108),
anti-CD31 FITC (clone 390, Biolegend #102405),
anti-Ter119 FITC (clone TER-119, Biolegend #116205),
anti-Sca1 Pacific Blue (clone D7, Biolegend #108119),
anti-PDGFRa PE (clone APA5 Biolegend #135905),
anti-CD3 APC (clone 17A2, Biolegend #100236),
anti-CD8 FITC (clone 53-6.7, Biolegend #100705),
anti-CD45 Pacific Blue (clone 30-F11, Biolegend #103126),
anti-CD4 PE (clone H129.19, Biolegend #130310),
anti-dsRNA antibody (J2, Jena Bioscience)

Western blot antibodies:
anti-IRF1 (D5E4) rabbit mAb (Cell Signaling #8478),
anti-pTBK (D52C2) rabbit mAb (Cell Signaling #5483),
anti-TBK (D1B4) rabbit mAb (Cell Signaling #3504),
anti-RIG-I (D14G6) rabbit mAb (Cell Signaling #3743), this antibody was also used for RIG-I immunoprecipitation.
anti-MDA5 (D74E4) rabbit mAb (Cell Signaling #5321),
anti-cytoskeletal actin (b-actin) rabbit polyclonal Ab (Bethyl cat# A300-485A),
Goat anti-rabbit IgG (H+L) secondary HRP-conjugated antibody (Thermo Fisher/Invitrogen #31460).

IF antibodies:
Goat anti-Mouse IgG (H+L) Secondary Antibody, Alexa Fluor Plus 488 (Thermo Fisher/Invitrogen #A32723)
anti-dsRNA antibody (J2, Jena Bioscience)
anti-CD3 (SP7) rabbit mAb (Novus Biologicals, #NB-600-1441)
anti-FoxP3 (D6O8R) rabbit mAb (Cell Signaling #12653)

Dilutions are provided in the manuscript.

Validation

All primary antibodies were confirmed on the species and application through the validation statement on the manufacturer's website and their use in the literature. We are providing here the list of antibodies used.
anti-CD45 FITC (clone 30-F11, Biolegend #103108), validated by the company (https://www.biolegend.com/en-us/products/fitc-anti-mouse-cd45-antibody-99), and by users (cited 116 times).
anti-CD31 FITC (clone 390, Biolegend #102405), validated by the company (https://www.biolegend.com/en-us/products/fitc-anti-mouse-cd31-antibody-120), and by users (cited 20 times).
anti-Ter119 FITC (clone TER-119, Biolegend #116205), validated by the company (https://www.biolegend.com/en-us/products/fitc-anti-mouse-ter-119-erythroid-cells-antibody-1865), and by users (cited 39 times).
anti-Sca1 Pacific Blue (clone D7, Biolegend #108119), validated by the company (https://www.biolegend.com/en-us/products/pacific-blue-anti-mouse-ly-6a-e-sca-1-antibody-3140), and by users (cited 45 times).
anti-PDGFRa PE (clone APA5 Biolegend #135905), validated by the company (https://www.biolegend.com/en-us/products/pe-anti-mouse-cd140a-antibody-6253), and by users (cited 35 times).
anti-CD3 APC (clone 17A2, Biolegend #100236), validated by the company (https://www.biolegend.com/en-us/products/apc-anti-mouse-cd3-antibody-8055), and by users (cited 68 times).
anti-CD8 FITC (clone 53-6.7, Biolegend #100705), validated by the company (https://www.biolegend.com/en-us/products/fitc-anti-mouse-cd8a-antibody-153), and by users (cited 116 times).
anti-CD45 Pacific Blue (clone 30-F11, Biolegend #103126), validated by the company (https://www.biolegend.com/en-us/products/pacific-blue-anti-mouse-cd45-antibody-3102), and by users (cited 113 times).
anti-CD4 PE (clone H129.19, Biolegend #130310), validated by the company (https://www.biolegend.com/en-us/products/pe-anti-mouse-cd4-antibody-5488), and by users (cited 2 times).
anti-dsRNA antibody (J2, Jena Bioscience), validated by the company (https://www.jenabioscience.com/rna-technologies/rna-analysis-detection/dsrna-detection/rnt-sci-10010-anti-dsrna-monoclonal-j2) and cited 11 times.
anti-IRF1 (D5E4) rabbit mAb (Cell Signaling #8478), validated by the company (https://www.cellsignal.com/products/primary-antibodies/irf-1-d5e4-xp-rabbit-mab/8478), and by users (cited 144 times).
anti-pTBK (D52C2) rabbit mAb (Cell Signaling #5483), validated by the company (https://www.cellsignal.com/products/primary-antibodies/phospho-tbk1-nak-ser172-d52c2-xp-rabbit-mab/5483), and by users (cited 509 times).
anti-TBK (D1B4) rabbit mAb (Cell Signaling #3504), validated by the company (https://www.cellsignal.com/products/primary-

antibodies/tbk1-nak-d1b4-rabbit-mab/3504), and by users (cited 322 times).
anti-RIG-I (D14G6) rabbit mAb (Cell Signaling #3743), validated by the company (https://www.cellsignal.com/products/primary-antibodies/rig-i-d14g6-rabbit-mab/3743), and by users (cited 150 times).
anti-MDA5 (D74E4) rabbit mAb (Cell Signaling #5321), validated by the company (https://www.cellsignal.com/products/primary-antibodies/mda-5-d74e4-rabbit-mab/5321), and by users (cited 112 times).
anti-cytoskeletal actin (b-actin) rabbit polyclonal Ab (Bethyl cat# A300-485A), validated by the company (https://www.fortislife.com/products/primary-antibodies/rabbit-anti-cytoskeletal-actin-antibody/BETHYL-A300-485), and by users (cited 15 times).
Goat anti-rabbit IgG (H+L) secondary HRP-conjugated antibody (Thermo Fisher/Invitrogen #31460). validated by the company (https://www.thermofisher.com/antibody/product/Goat-anti-Rabbit-IgG-H-L-Secondary-Antibody-Polyclonal/31460), and by users (cited 1929 times).
anti-FoxP3 (D6O8R) rabbit mAb (Cell Signaling #12653), validated by the company (https://www.cellsignal.com/products/primary-antibodies/foxp3-d6o8r-rabbit-mab/12653), and by users (cited 63 times).
anti-CD3 (SP7) rabbit mAb (Novus Biologicals, #NB-600-1441), validated by the company (https://www.novusbio.com/products/cd3-antibody-sp7_nb600-1441), and by users (cited 27 times).

Eukaryotic cell lines

Policy information about cell lines and Sex and Gender in Research

Cell line source(s)	HEK 293T cells were purchased from ATCC.
Authentication	No other independent authentication was performed.
Mycoplasma contamination	HEK 293T tested negative for mycoplasma contamination, and the cells were kept under prophylactic Plasmocin treatment during experiments (InvivoGen).
Commonly misidentified lines (See ICLAC register)	None of these cell lines were used in this study.

Animals and other research organisms

Policy information about studies involving animals; ARRIVE guidelines recommended for reporting animal research, and Sex and Gender in Research

Laboratory animals	Wild type mice (strain #000664) and p53KO mice (#002101) were purchased from The Jackson Laboratory. With the exception of athymic nude mice (#007850), all the experimental animals were kept in C57BL/6 background. Females (12 weeks old to 6 months old) were maintained and used for breeding purposes. Mice were maintained in ambient room temperature (22 +/- 2 C) with humidity of 40-70% and light cycle of 12/12h. Maximal tumor burden and all other aspects of animal experiments were performed in accordance with the guidelines of Cedars-Sinai Medical Center Institutional Animal Care and Use Committee.
Wild animals	This study did not involve wild animals.
Reporting on sex	Sarcoma cells were generated from female mice, and tumor recipients were females.
Field-collected samples	This study did not involve field collected samples.
Ethics oversight	Animal experiments were performed in accordance with the guidelines of Cedars-Sinai Medical Center Institutional Animal Care and Use Committee.

Note that full information on the approval of the study protocol must also be provided in the manuscript.

Flow Cytometry

Plots

Confirm that:

☒ The axis labels state the marker and fluorochrome used (e.g. CD4-FITC).

☒ The axis scales are clearly visible. Include numbers along axes only for bottom left plot of group (a 'group' is an analysis of identical markers).

☒ All plots are contour plots with outliers or pseudocolor plots.

☒ A numerical value for number of cells or percentage (with statistics) is provided.

Methodology

Sample preparation	Tumors were dissociated by enzymatic digestion with the OctoMacs (Miltenyi) to single cell suspension. Cells were filtered twice through 70 µm filters. Red blood cells were lysed with ACK solution (Gibco). After ACK, cells were washed twice with PBS, and then stained with the fluorophore-conjugated antibodies for 15 minutes at room temperature. The excess of unbound antibodies was washed out before acquisition in flow cytometry.

Instrument	BD FACSAria and BD LSR II.
Software	Flow cytometry data was collected with BD FACS Diva 8.0.3 software and analyzed with FlowJo v10.
Cell population abundance	Purity of the sorted cells was not assessed post-sorting due to the limited numbers of sorted cells. In addition, the markers assessed would not be expressed by any other cell types potentially present. Total RNA was extracted from the sorted populations and normalized before RTqPCR.
Gating strategy	Forward scatter vs. side scatter plot was used to separate cell events from debris. Non-stained negative controls were used in the experiments to define gating.

☒ Tick this box to confirm that a figure exemplifying the gating strategy is provided in the Supplementary Information.
